# Biodegradable Polymers for Plant Nutrient Delivery and Recovery

**DOI:** 10.1002/mabi.202500042

**Published:** 2025-03-25

**Authors:** Alice Boarino, Nicola Carrara, Elio Padoan, Luisella Celi, Harm‐Anton Klok

**Affiliations:** ^1^ Department of Agricultural Forest and Food Sciences University of Turin Largo Paolo Braccini 2 Grugliasco 10095 Italy; ^2^ Institut des Matériaux and Institut des Sciences et Ingénierie Chimiques Laboratoire des Polymères École Polytechnique Fédérale de Lausanne (EPFL) Station 12 Lausanne 1015 Switzerland

**Keywords:** adsorbents, flocculants, hydrogels, nanofertilizers, polymer membranes, slow‐ and controlled‐release fertilizers

## Abstract

The current use of fertilizers is inefficient and not sustainable. The majority of the fertilizer applied does not reach the targeted crop but is lost in the water bodies and into the atmosphere, with harmful impact on the environment. To enhance the efficiency and sustainability of current agricultural practices, it is essential to address two complementary challenges. First, nutrient delivery methods must be refined to maximize plant uptake. Second, the recovery of nutrients from wastewater and other waste streams should be improved to enhance the recycling of nitrogen and phosphorous and reduce environmental pollution. Biodegradable polymers hold great promise for the development of technological solutions toward more sustainable agricultural practices. This review covers the application of biodegradable polymers in both aspects of the nutrient cycle: nutrient delivery to plants through slow‐ and controlled‐release fertilizers, and nutrient recovery from wastewater using membrane separation, adsorbent composites, and coagulants/flocculants. The most promising materials are highlighted for both approaches, identifying the research gaps and discussing potential future directions in this highly significant field.

## Graphical abstract

This review article discusses the use of biodegradable polymers to address several grand challenges in agriculture: to improve the delivery of nitrogen and phosphorous to crops, to optimize plant uptake, and to enhance the recovery of these nutrients from wastewater, to recycle them and reduce water pollution.

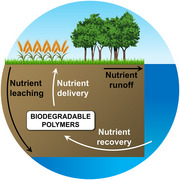

## 1 Introduction

The most pressing challenges to ensure peace and prosperity for people all over the world are outlined in 17 sustainable development goals (SDGs), defined in the 2030 Agenda for Sustainable Development adopted by the United Nations in 2015.^[^ ^1^ ^]^ Agriculture is a key sector that influences many aspects of sustainability, from food security and economic growth to environmental conservation and climate change mitigation, and agricultural practices act as a common link to all SDGs.^[^ ^2^ ^]^ Fertilizers are essential to provide food to the growing world population. In 2020, the worldwide annual consumption of nitrogen (N) and phosphorous (P) fertilizers has been 120×10^6^ and 50×10^6^ metric tonnes, respectively. Without a decisive change in the current agricultural practices, the environmental impact of this massive consumption is estimated to increase 50–90% by 2050, including the use of water and cropland, as well as the emission of greenhouse gases (GHG).^[^ ^3^ ^]^ The current use of these nutrients in plant fertilization is very inefficient.^[^ ^4^, ^5^ ^]^ Only 30–55% of the N and 18–20% of the P applied to the soil is reaching the targeted crop, while the rest is lost via leaching, run off, and volatilization.^[^ ^6^, ^7^, ^8^, ^9^ ^]^ Not only is this a huge waste of resources, but it also causes negative effects on the environment, such as eutrophication and water contamination, as well as air contamination and global warming.^[^ ^7^, ^10^ ^]^ To improve the efficiency and the circularity of the current agricultural practices, it is fundamental to tackle two complementary challenges. On the one hand, nutrient delivery techniques must be improved to optimize plant uptake. On the other hand, nutrient recovery from wastewater and other waste streams must be improved to reuse N and P and reduce environmental pollution. Wastewater is both a major responsible of environmental contamination and a crucial source from which nutrients can be recovered.^[^ ^11^, ^12^ ^]^ This is particularly important for N and P because the production of fertilizers based on these two elements is energy intensive and contributes to global greenhouse gas emissions. Nitrogen fertilizers are produced via the Haber–Bosch process, responsible for 1.4% of carbon dioxide emissions worldwide and consuming 2% of the global energy production.^[^ ^13^, ^14^, ^15^ ^]^ Phosphorous production is concentrated in only three main countries: USA, China, and Morocco.^[^ ^16^, ^17^ ^]^ Due to its great importance and high supply risk, the European Commission has classified phosphate rock as one of the 20 Critical Raw Materials.^[^ ^18^ ^]^ Therefore, recovery is not only important to mitigate environmental damage, but also to allow the recovery and reuse of essential and finite resources.

**Figure 1:**
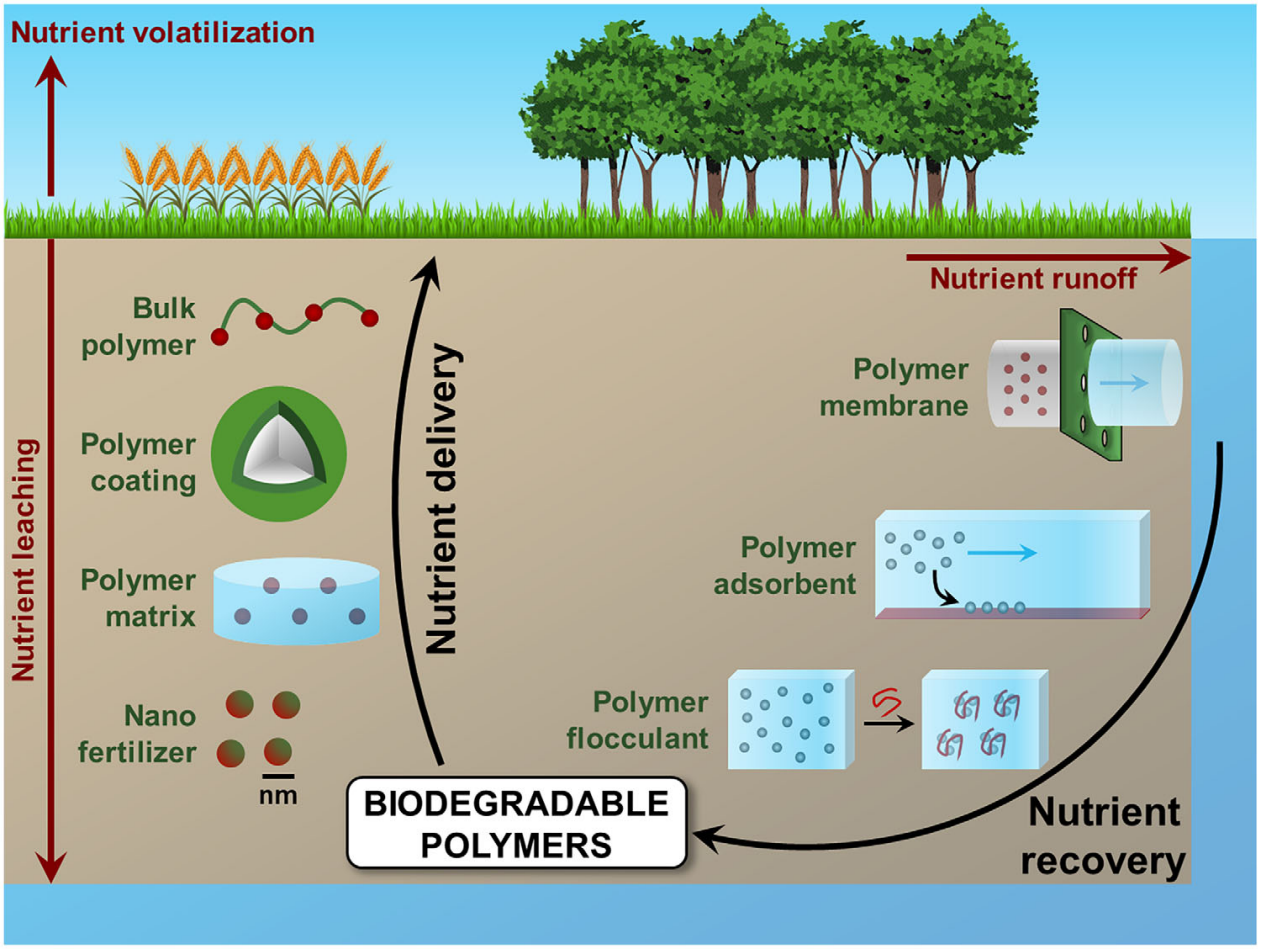
Schematic illustration of the use of biodegradable polymers to enhance nutrient delivery and nutrient recovery in agriculture.

Biodegradable polymers are ideal candidates for the development of technological solutions that will help to improve nutrient delivery and recovery (**Figure** **1**).^[^ ^19^, ^20^, ^21^ ^]^ Unlike traditional plastics, which can persist for centuries contributing to soil pollution, biodegradable polymers can deteriorate in soil over timeframes that can vary from months to years, depending on, for example, the composition and molecular weight of the polymer, as well as environmental conditions such as temperature, humidity, and microbial presence.^[^ ^22^, ^23^, ^24^, ^25^ ^]^ When biodegradable polymers are completely converted into CO_2_ and water, their environmental impact is significantly reduced, making them a more sustainable alternative for reducing plastic waste and pollution in soil and ecosystems.^[^ ^23^, ^26^, ^27^ ^]^ Polymer degradation in soil is regulated by the microbiome.^[^ ^28^, ^29^ ^]^ The soil microorganisms colonize the material surface and produce extracellular enzymes that hydrolyze the polymer chain into smaller molecules. The products of enzymatic hydrolysis are then absorbed by the plants or consumed by the microorganisms as a source of carbon and energy, completing the biodegradation process. The complete step‐by‐step mechanism of polymer degradation in the soil ecosystem has been described in detail by Mo et al.^[^ ^26^ ^]^

Biodegradable polymers applied in agriculture can be classified as natural, like cellulose, chitosan, starch, alginate, and lignin, and synthetic, like poly(lactic acid) (PLA), poly(vinyl alcohol) (PVA), and poly(acrylic acid) (PAA).^[^ ^20^, ^26^ ^]^ There has been significant interest to explore these materials for the development of slow‐ and controlled‐release fertilizers.^[^ ^8^, ^30^, ^31^, ^32^, ^33^, ^34^, ^35^ ^]^ By limiting and controlling the rate of nutrient release, this technology can enhance nutrient use efficiency and reduce nutrient losses in the environment. Polymers can be used in a number of ways to generate slow‐ and controlled‐release fertilizers. A first approach involves polymers that can act as fertilizers themselves by gradual release of nutrients upon degradation of the polymer. A second class of slow‐/controlled‐release systems is composed of nutrient granules coated with a polymer film. Finally, polymer‐based slow‐/controlled‐release fertilizers can be obtained by incorporating nutrients in a polymer matrix, or by preparing nanosized formulations of polymers and plant nutrients (Figure 1). Biodegradable polymers are also important components for the development of strategies for nutrient recovery. Examples include the use of biodegradable polymers in membrane separation, adsorbent composites and coagulants/flocculants for nutrient recovery from wastewater. A wide number of articles and reviews have already been published, typically focusing on only one side of the coin: either on fertilizer delivery, or on nutrient removal from wastewater. In addition, reviews about wastewater treatment usually focus on the removal of contaminants, not on the recovery of nutrients. An overview of the opportunities provided by biodegradable polymers for the recovery of N and P seems timely and of high interest to a wide readership. Herein we consider both sides of the nutrient cycle, delivery to plant and recovery from wastewater, highlighting the most promising polymers for both approaches, and pointing out the research opportunities and possible future prospectives.

## 2 Biodegradable Polymers for Nutrient Delivery

One approach to improve nutrient delivery to plants is the application of slow‐ or controlled‐release fertilizers, which supply the nutritive substance over a longer period than conventional mineral fertilizers. By providing N and P to the plant over a wider time frame, this technology can enhance the nutrient use efficiency, reduce the need for repeated fertilization, minimize the risk of nutrient losses in the environment, and help to avoid water contamination.^[^ ^9^, ^30^ ^]^ Slow‐release fertilizers deliver the nutrient more slowly than conventional ones, where the nutrient is immediately available for the plant, but the delivery rate only depends on soil and climatic conditions and cannot be precisely predicted and controlled. Controlled‐release fertilizers are defined as such when the factors regulating the duration and rate of nutrient release follow a well‐defined pattern.^[^ ^30^, ^36^, ^37^ ^]^

**Figure 2:**
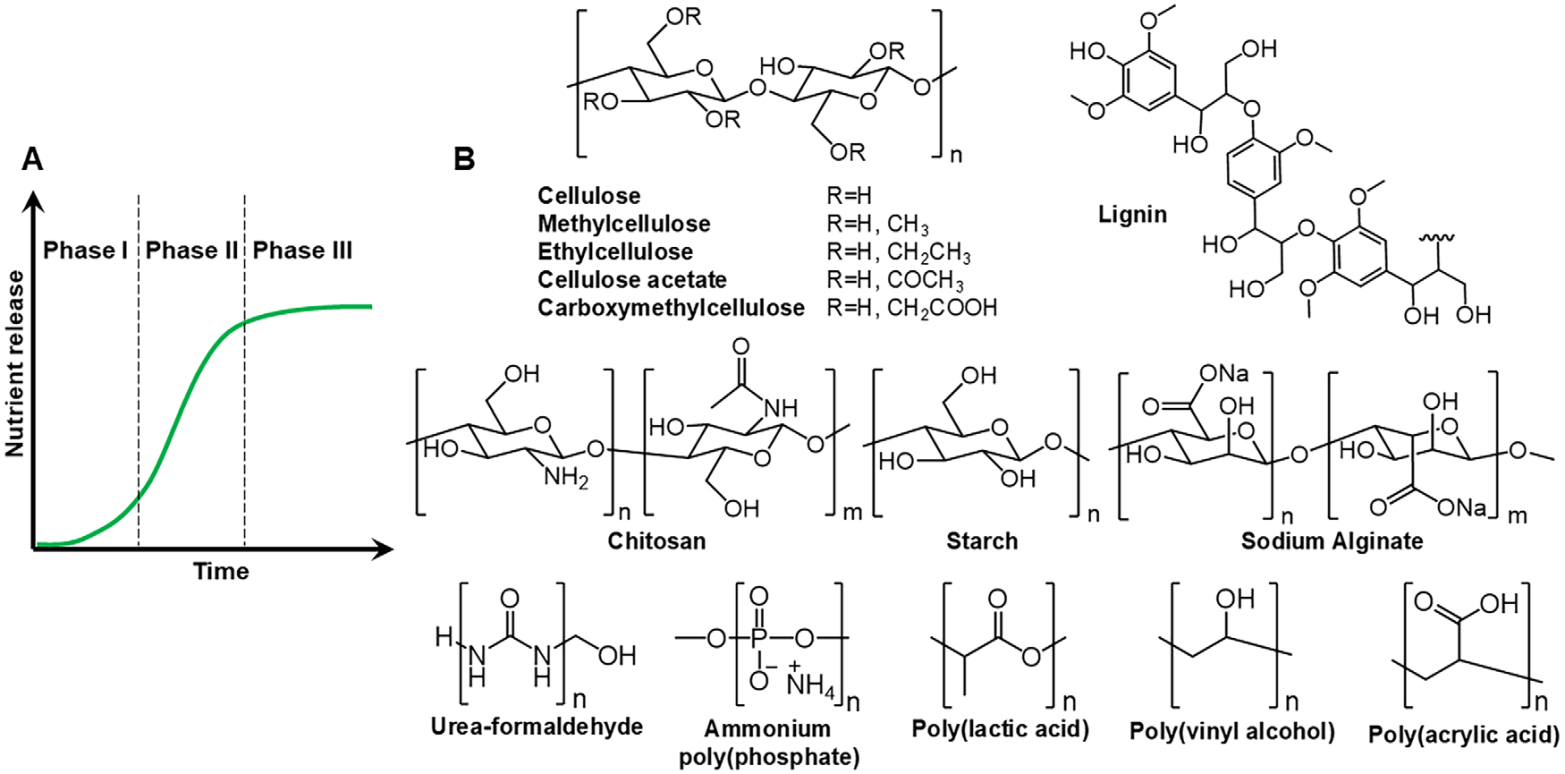
A) Release rate of nutrients from the controlled‐release fertilizers, matching the crop nutrient uptake rate. B) Chemical structure of the biodegradable polymers used for the preparation of slow‐ or controlled‐release fertilizers.

Ideally, the nutrient release pattern from the fertilizer should match the crop nutrient uptake rate. Trenkel describes this pattern as a sigmoidal curve consisting of three phases (**Figure** **2A**).^[^ ^36^ ^]^ In the first one, called lag phase, soil moisture penetrates the insoluble fertilizer coating or matrix driven by the water vapor gradient. In the second phase, the water inside the insoluble barrier solubilizes the solid nutrient and allows it to be gradually released through cracks in the coating or the swollen protective shell, at a constant rate. In the third phase, the insoluble coating or matrix breaks due to the water pressure, completely releasing the cargo. The delivery rate in this phase is lower than in the previous one, since most of the nutrients have already been released previously.^[^ ^36^, ^37^ ^]^ In addition, many factors can influence the nutrient release, which are both related to soil conditions (e.g. texture, moisture, pH, soil organic matter and microbiome), as well as to materials characteristics of the fertilizer such as hydrophobicity, size and mechanical properties.^[^ ^37^ ^]^ The latter are important in view of the handling, transportation, storage, and application of the fertilizer, especially when in form of granules and pellets. Granules holding high mechanical strength are preferred because they are less likely to dust and degrade during storage, shipment or distribution, reducing losses and risk of uneven spread in the soil.^[^ ^38^, ^39^ ^]^

Polymers are ideal materials to prepare slow‐ and controlled‐release fertilizers.^[^ ^19^, ^33^ ^]^ Oil‐derived polymers like polyethylene, polypropylene, polystyrene and poly(vinyl chloride) have been widely applied with this purpose, thanks to their versatility and tunable properties.^[^ ^19^, ^40^ ^]^ Although these polymers can efficiently slow down the release of plant nutrients, the majority of them is not degradable and can remain in soil for years causing environmental pollution. Biodegradable polymers can overcome this issue, providing a sustainable alternative to petroleum‐based ones. Examples of biodegradable polymers that have been used for the preparation of slow‐ and controlled‐release fertilizers are shown in Figure 2B, and include natural biodegradable polymers, such as for example cellulose,^[^ ^41^, ^42^, ^43^ ^]^ chitosan,^[^ ^44^, ^45^ ^]^ starch,^[^ ^35^ ^]^ alginate,^[^ ^46^, ^47^, ^48^ ^]^ and lignin,^[^ ^31^, ^49^, ^50^, ^51^ ^]^ as well as synthetic biodegradable polymers such as poly(lactic acid), poly(vinyl alcohol) and poly(acrylic acid).^[^ ^32^, ^33^, ^37^, ^40^ ^]^

The following sections will present the use of biodegradable polymers toward the preparation of slow‐ and controlled‐release fertilizers.

### 2.1 Biodegradable Polymers for Slow‐ and Controlled‐Release Fertilizers

One strategy for developing slow‐ and controlled‐release fertilizers involves the use of polymers, which release nutrients upon microbial and/or abiotic hydrolysis/degradation in soil (**Table** **1**). The release is controlled by both environmental parameters (e.g., microbial activity, temperature and pH) and structure and composition of the polymer.^[^ ^36^ ^]^

**Table 1:** Biodegradable polymers utilized for slow‐ and controlled‐release fertilizers.

Polymer	Nutrient	Nutrient release in water or [soil]	Ref.
Urea‐formaldehyde	N	[few weeks to 1–2 years]	[52]
Urea‐formaldehyde	N	20–80% after 50 h [<25% after 42 d]	[53]
Urea‐formaldehyde	N	–	[54, 55]
Ammonium poly(phosphate)	N, P	7.85–9.20% in 80 d at pH 6	[56]
Ammonium poly(phosphate)/Poly(phosphoric acid)	N, P	9.8–31% after 100 d at pH 5 and 25 °C	[57]
Ammonium poly(phosphate)	N, P	–	[58]
Ammonium poly(phosphate)	N, P	–	[59]
Ammonium poly(phosphate)	N, P	<40% in 50 h at 60 °C at pH 6	[60]
Poly(methylene‑co‑cyanoguanidine)	N	–	[61]
Aminated Lignin	N	<10% in 28 d^a^ ^)^	[62]

^a)^ Calculated based on the data provided in the study and subtracting the N released from the control.

One example is represented by urea‐based slow‐release polymer fertilizers, which have been studied and made commercially available since the 1950s. These fertilizers, produced by condensing urea and formaldehyde (UF), were the first slow‐release nitrogen fertilizers introduced. Commercial production began in 1955, and they remain widely used today, despite their high production costs limiting broader market adoption.^[^ ^36^, ^54^ ^]^

Over time, various regulations and guidelines have been established to promote the optimal use of urea‐formaldehyde‐based slow‐release fertilizers,^[^ ^36^, ^63^ ^]^ while studies have examined their potential environmental and health toxicity, particularly due to the release of formaldehyde.^[^ ^64^ ^]^ Most of the research nowadays is focused on production process optimization and modelling to obtain the best urea‐formaldehyde formulation. For instance, Giroto et al. investigated a way to produce urea‐formaldehyde with delivery times suitable for crop plants.^[^ ^53^ ^]^ They produced a partially polymerized urea‐formaldehyde granule, different from the standard fully polymerized available products, via a simple melt polymerization process and suitable for large‐scale production. The final product properties and N release profiles could be controlled by finely tuning the urea, paraformaldehyde and water ratios in the production process. Guo et al. dedicated their attention to the mathematical modelling of the synthetic process of urea‐formaldehyde fertilizers using response surface methodology to find the optimal parameters (e.g., temperature, pH, urea/formaldehyde ratios) to maximize the cold water insoluble fraction and minimize the hot water insoluble fraction.^[^ ^54^, ^55^ ^]^ This led to formulations with intermediate delivery times, suitable for crop needs.

Right after the development of urea‐formaldehyde, starting from the ‘60s another polymeric formulation was developed for P, the second most important macronutrient. Polyphosphate‐based products, and especially ammonium‐polyphosphates, were developed to improve the P use efficiency compared to orthophosphate‐based fertilizers (e.g., ammonium phosphates, calcium phosphates), since orthophosphate ions are slowly released through the progressive hydrolysis of the polyphosphate chains taking place in soil. This process increases the availability of P to plants during the plant life cycle and reduces its inaccessibility via retention processes such as adsorption and precipitation. The efficacy of orthophosphate‐based fertilizers is extremely low and only a small percentage (<20%) is absorbed by plant.^[^ ^6^ ^]^ Inorganic phosphate easily forms stable complexes with metal cations (e.g., Al^3+^, Fe^2+/3+^) and clay components, or can be adsorbed on positively charged mineral surfaces such as Fe and Al (hydr)oxides, reducing its mobility and availability to plants, which mainly uptake P in the forms of HPO_4_^2−^ and H_2_PO_4_^−^.^[^ ^65^ ^]^ The P‐release kinetics of ammonium polyphosphate are complex, depending on the intrinsic properties of the products as well as on biotic and abiotic parameters.^[^ ^57^, ^60^, ^65^ ^]^ Yang et al. synthesized ammonium polyphosphate *in‐situ* reacting mono‐ammonium phosphate and urea, as a condensation agent, in a twin‐screw extruder.^[^ ^56^ ^]^ The heterogeneity of the sample allowed for progressive hydrolysis over time, maintaining a constant level of available P. Wang et al. highlighted important aspects underlying the hydrolysis process of polyphosphates both in water and in soil, as well as the ability of this material to chelate micronutrients, such as Fe, Mn, Cu and Zn.^[^ ^57^ ^]^ The authors observed that the release of orthophosphate ions from the short‐chain polyphosphates is 2.7 times higher at 35 °C compared to 25 °C in water and 1.12 times higher in soil, measured as Olsen‐P (measured using the Olsen method, quantifying the plant‐available inorganic P in soil).^[^ ^66^ ^]^ Alkaline soil favored a higher hydrolysis rate compared to acidic soil, differently from the trend observed in *in vitro* tests. Their results also pointed out the importance of enzyme phosphatases in the polyphosphates degradation in soil. In addition, the application of polyphosphate fertilizers to calcareous soil increased P and micronutrient availability compared to the orthophosphate‐based formulation. It has been observed that the interaction between metal cations and oxides and polyphosphate chains is rather complex. Various studies have demonstrated that the presence of micronutrients such as Zn^2+[^ ^60^ ^]^ and Fe (hydr)oxides^[^ ^67^ ^]^ can influence the hydrolysis of polyphosphates. Maize growth using ammonium polyphosphate as a P fertilizer showed significantly higher P and micronutrient uptake compared to monoammonium phosphate fertilization.^[^ ^58^ ^]^ Importantly, polyphosphate‐based products demonstrated their higher efficacy, showcasing a P use efficiency of 43% compared to 25% of the monoammonium phosphate. In addition, the results suggested that a higher degree of polymerization (3.8 *vs* 3) and a higher polyphosphate/total P ratio (90% *vs* 70%) could be beneficial for crop growth. Positive results on the use of ammonium polyphosphate as P fertilizer were obtained by Kourchi et al.; the authors observed that durum wheat grown under ammonium polyphosphate fertilization favoured P uptake, photosynthesis and growth performance compared to the control.^[^ ^59^ ^]^ In addition, they investigated the role of the rhizosphere environment and microbiome in stimulating the degradation of polyphosphate chains through the exudation of phosphatases and organic acids. Along this line, they showed that co‐applying ammonium polyphosphate fertilizer and P‐solubilizing bacteria enhanced the available P to the plant, leading to improved P uptake and wheat growth.^[^ ^68^ ^]^

Very few other polymeric fertilizers have been developed and investigated. Rychter et al. evaluated the use of poly(methylene‐*co*‐cyanoguanidine) as a novel slow‐release nitrogen fertilizer.^[^ ^61^ ^]^ Laboratory plant experiments performed on oat and radish demonstrated growth promotion on both tested plants. In addition, ecotoxicological assessments indicated that the material is both biocompatible and non‐toxic.

A different approach was utilized to develop slow‐ and controlled‐release fertilizers based on lignin, the most abundant aromatic polymer in nature.^[^ ^49^, ^51^ ^]^ Lignin is found in the plant cell wall, where it provides mechanical strength and support to the plant.^[^ ^50^, ^69^ ^]^ This material is considered a byproduct of the chemical pulping processes and biomass‐based ethanol production, and hence discarded in huge quantities to be mainly burned as low value fuel. Its employment as fertilizer material is therefore advantageous from the economic and sustainability points of view.^[^ ^31^ ^]^ Being a precursor of humic substances, lignin favors soil remediation and the phenolic products of its degradation function as urease inhibitors, reducing nitrification and volatilization.^[^ ^70^, ^71^ ^]^ Lignin can be enriched in N via Mannich reaction or ammoxidation. Jiao et al. attached to lignin various reagents containing amine groups through the Mannich reaction.^[^ ^62^ ^]^ The authors found out that the main factor influencing the N content in the final product was the type of amination reagent, and that the aminated lignin was able to release ammonium and nitrate at a slower rate than pure urea.

### 2.2 Biodegradable Polymers as Coatings for Slow‐ and Controlled‐Release Fertilizers

In this type of fertilizer, the water‐soluble nutrient in form of granules, beads, or particles with sizes of typically 2–4 mm is coated by a hydrophilic or hydrophobic polymer layer.^[^ ^72^ ^]^ In slow‐release fertilizers, the nutritive substance is gradually delivered by diffusion through the insoluble layer. Controlled‐release fertilizers can be designed to respond to more specific stimuli, like pH, temperature, and interaction with enzymes and microorganisms. Common processes for the preparation of coated fertilizers are spray coating, pan coating, and fluidized bed coating.^[^ ^40^, ^73^, ^74^ ^]^ Alternatively, the nutrient can be encapsulated in the insoluble shell by crosslinking^[^ ^75^, ^76^, ^77^ ^]^ or polymerization.^[^ ^78^, ^79^, ^80^, ^81^ ^]^

#### 2.2.1 Natural Biodegradable Polymers as Coatings for Slow‐ and Controlled‐Release Fertilizers

Natural polymers present many advantages for the application in agriculture, including wide availability, low cost, biocompatibility and biodegradability.^[^ ^82^ ^]^ An overview of natural biodegradable polymers utilized as coating material in slow‐ and controlled‐release fertilizers is presented in **Table** **2**.

**Table 2:** Examples of natural biodegradable polymers utilized as coatings in slow‐ and controlled‐release fertilizers.

Polymer	Particle diameter	Nutrient	Nutrient release in water or [soil]	Ref.
Carboxymethyl cellulose/hydroxyethyl cellulose + cellulose particles	2–4 mm	N, P	12 h [19–20 d]	[83]
Ethylcellulose	2–5 mm	N, P, K	<75% in 28 d	[84]
Methylcellulose/lignin	3–4 mm	N, P	100% in 30 d	[85]
Chitosan	0.3–0.4 mm	N, P, K	64% N, 14% P, 41% K in 30 d	[86]
Chitosan/poly(acrylic acid)	2.52–3.54 mm	N, P, K	[75% in 30 d]	[87]
Starch/poly(acrylic acid)/natural rubber/poly(acrylic acid)	2–4 mm	N	47.5% in 168 h, [38.5% in 30 d]	[88]
Starch/poly(acrylic acid) + Fe^3+^	2–5 mm	N	[52% in 18 d]	[77]
Sodium alginate	≈ 3 mm	N	[94.2% in 25 d]	[46]
Sodium alginate + SiO_2_	≈ 3 mm	N	15% in 24 h, 56% in 28 d, 100% in 60 d	[48]
Sodium alginate/lignin/carboxymethyl cellulose	2–3 mm	P	59.5% in 3 d	[89]
Lignin	3–4 mm	N	86.9% in 44 d	[90]
Lignin	≈ 2 mm	P	20–50% in 4 d	[91]
Lignin/sodium alginate	2–4 mm	N, P	100% in 30 d	[92]
Lignin/sodium alginate/poly(acrylic acid)	1–3 mm	N	43% in 5 d, 72% in 10 d	[93]
Lignin/poly(acrylic acid)/ poly(acrylamide)	1–5 mm	N, P, K	5.15% in 2 d, 59.51% in 28 d	[94]

Cellulose is the polysaccharide most widely distributed in nature, found in wood, cotton, hemp, bagasse and many other plants. This biodegradable polymer is hydrophilic but insoluble in water.^[^ ^42^, ^43^ ^]^ For the application in agriculture, cellulose can be used as it is, or modified by etherification and esterification, forming cellulose derivatives with slightly different properties than the original polymer. Kassem et al., for instance, have coated monoammonium phosphate with a mixture of different cellulose derivatives, including carboxymethyl cellulose (CMC), hydroxyethyl cellulose and spherical regenerated cellulose particles using a simple rotating pan method.^[^ ^83^ ^]^ The use of coated fertilizer allowed to prolong release to 12 h in water and 19–20 days in soil, as compared to 1–2 h in water and 9–10 days in soil of uncoated fertilizer. In another study, published by Lubkowski et al., ethylcellulose has been used to coat NPK granules via immersion technique.^[^ ^84^ ^]^ The authors utilized different mass ratios of polymer:fertilizer (0.165–0.285), achieving a variable layer thickness (204–244 µm). The coated granules displayed significantly better mechanical properties in comparison with the pure fertilizer. The samples prepared with ethylcellulose:NPK ratio higher than 0.21 released less than 75% P in water within 28 days, outperforming the uncoated fertilizer, completely solubilized in 1 h. El Bouchtaoui et al. utilized methylcellulose crosslinked with lignin as a coating material to encapsulate ammonium phosphate and triple superphosphate.^[^ ^85^ ^]^ By changing the ratio between methylcellulose and lignin in the coating, the authors optimized the hydrophobicity and mechanical properties of the composite, as well as the nutrient release rate. The coating with the highest lignin content (40%wt.) allowed the slowest N and P release, which terminated after 30 days.

Another linear polysaccharide widely applied in slow‐ and controlled‐release fertilizers is chitosan, derived by deacetylation of chitin, the second most abundant natural compound after cellulose.^[^ ^95^ ^]^ Chitosan is hydrophilic and shows a variety of attractive properties, including film forming ability, chelating capacity and presence of reactive groups for chemical activation and crosslinking.^[^ ^44^, ^45^ ^]^ The presence of amino groups along the chitosan chains allows for chemical modifications, and the protonation of the amino group under acidic conditions favors electrostatic interactions with negatively charged molecules and a pH‐responsive behavior. Chitosan has been used to coat nutrients and delay their release alone^[^ ^86^ ^]^ or in the presence of crosslinkers such as tripolyphosphate. Gumelar et al. have sprayed a pure chitosan solution on NPK in a rotary pan granulator, achieving coated granules that released only 64% N, 14% P, and 41% K after 30 days, significantly more slowly than the uncoated fertilizer.^[^ ^86^ ^]^ Wu et al. coated NPK granules with an inner chitosan coating and then applied a second outer layer of poly(acrylic acid)/diatomite containing urea.^[^ ^87^ ^]^ This double‐coated fertilizer showed a slow release of nutrients in soil, not exceeding 75% over 30 days.

Starch is another largely abundant biopolymer present in the leaves, fruit, and roots of most crops.^[^ ^37^ ^]^ The ability of this polymer to act as a nutrient carrier has been widely employed to prepare slow‐ and controlled‐release fertilizers, due to its large availability, low cost, biodegradability and biocompatibility.^[^ ^35^ ^]^ Nutrients can be either physically or chemically incorporated into a starch coating, but due to its poor mechanical resistance and plasticity, starch is normally used in combination with other materials. Tanan et al. coated urea with a hydrogel made of starch, PAA, natural rubber and PVA.^[^ ^88^ ^]^ The authors were able to tune the coating swelling, water retention and biodegradability by changing the ratio between natural rubber and PVA, achieving a N release rate of 47.5% in 168 h in water and 38.5% over 30 days in soil. Xu et al. prepared a hydrogel by grafting and mixing starch with poly(acrylic acid) and urea, which was improved by ionic crosslinking with Fe^3+^.^[^ ^77^ ^]^ The achieved coating could delay urea release from >90% to 52% over 18 days.

Another biodegradable polymer widely used in agriculture is alginate, which can be extracted from marine algae.^[^ ^96^ ^]^ This material, characterized by good biocompatibility and biodegradability, can easily crosslink with bivalent cations and form beads incorporating a desired nutrient.^[^ ^40^, ^97^ ^]^ Wang et al. coated urea with an inner layer of sodium alginate and an outer layer of poly(acrylic acid)/celite superabsorbent, achieving a N release in soil of 94.2% in 25 days.^[^ ^46^ ^]^ De Matos et al. incorporated ammonium nitrate inside an alginate matrix containing biogenic silica, a filler able to control the coating porosity and thus the N release rate from the granules.^[^ ^48^ ^]^ Sodium alginate was crosslinked with calcium ions forming a stable polymeric network, in which the fertilizer and 1–10% biogenic silica was incorporated. The nitrogen release rate was controllable changing the content of SiO_2_ in the coating, prolonging the release over 60 days. Fertahi et al. have coated triple superphosphate granules with a composite made of sodium alginate, k‐carrageenan, carboxymethyl cellulose and lignin.^[^ ^89^ ^]^ The P release was delayed from 100% within 48 h for the uncoated granules to 60% within 72 h for the formulation 5:1 fertilizer:polymer.

Lignin has also been used as coating material to control nutrient release. Wei et al. synthesized a lignin coating showing reduced urea release to 86.9% over 44 days.^[^ ^90^ ^]^ The coating was obtained by first crosslinking lignin with epichlorohydrin, and then esterification with lauryl chloride. Lignin has also been employed to coat and deliver P, as shown in a study by Rotondo et al.^[^ ^91^ ^]^ The coating used in this study was obtained by hydroxymethylating lignin, followed by crosslinking with a phenol‐formaldehyde resin and was applied to the surface of 2 mm size superphosphate granules by means of a heated pan coater. El Bouchtaoui et al. formed a uniform polymeric film made of lignin and sodium alginate capable of incorporating ammonium phosphate and reducing its dissolution rate.^[^ ^92^ ^]^ An increase of lignin content in the coating (from 25%wt. to 75%wt.) corresponded to a slower N and P release. The nutrients were completely released only after a month, way later than the uncoated fertilizer, available in only four days. Zhang et al. prepared a double hydrogel‐based coating with the ability to regulate N release from urea granules, achieving 43% delivery in 5 days and 72% in 10 days.^[^ ^93^ ^]^ The inner shell was realized incorporating nanoparticles made of lignin and bentonite in an alginate matrix, while the outer layer was made of poly(acrylic acid). Li et al. produced a superabsorbent polymer outer coating for urea granules based on attapulgite‐doped polyacrylic acid and polyacrylamide grafted alkali lignin.^[^ ^94^ ^]^ The final product efficiently managed to reduce urea release (59.5% released after 28 days) displaying an optimal water retention capacity (up to 240 g/g).

#### 2.2.2 Synthetic Biodegradable Polymers as Coatings for Slow‐ and Controlled‐Release Fertilizers

An overview of synthetic biodegradable polymers that are used as coatings slow‐ and controlled‐release fertilizers is provided in **Table** **3**.

**Table 3:** Synthetic biodegradable polymers utilized as coating in slow‐ and controlled‐release fertilizers.

Polymer	Particle diameter	Nutrient	Nutrient release in water or [soil]	Ref.
Poly(lactic acid)	50–100 µm	N	0.5% in 30 d	[98]
Poly(lactic acid)/cellulose acetate	4 mm	N, P	24.9% in 24 h	[99]
Poly(ethyl glyoxylate)/poly(caprolactone) or poly(lactic acid)	4.3 mm	N	100% in 21d at 30 °C, 20% in 21d at 22 °C	[100]
Poly(lactic acid)/poly(caprolactone)	2 mm	N	[13% in 1d, 81% in 28 d]	[101]
Poly(acrylic acid)	–	N, P	56% P, 28% N in 28 d	[102]
Poly(acrylic acid)	2–5 mm	N	≈ 60% in 30 d	[103]
Poly(vinyl alcohol)/chitosan/poly(acrylic acid‐*co*‐acrylamide)	1–4 mm	N, P, K	84% N, 63% P, 36% K in 30 d	[104]
Poly(vinyl alcohol)/poly(lactic acid)	0.2–0.5 µm	N, P, K	90% in 90 d	[105]

Poly(lactic acid) is a hydrophobic polymer, which can be derived from agricultural products.^[^ ^106^, ^107^ ^]^ Thanks to its biocompatibility, thermoplasticity and strength, it is widely employed in packaging and biomedical applications, but a large number of studies have recently focused also on its employment for agricultural purposes.^[^ ^33^, ^75^ ^]^ Papangkorn et al., for instance, prepared microparticles of urea coated with PLA, which released only 0.1% N in 2 weeks and 0.5% after 30 days.^[^ ^98^ ^]^ El Assimi et al. encapsulated granules of diammonium phosphate in a coating of PLA and cellulose acetate using a rotary pan.^[^ ^99^ ^]^ The addition of cellulose acetate reduced the crystallinity of poly(lactic acid), and accelerated the biodegradation of the coating in soil. Heuchan et al. have coated urea granules with poly(ethyl glyoxylate) blended with poly(caprolactone) or PLA.^[^ ^100^ ^]^ The coatings with poly(caprolactone) were responsive to pH and temperature, showing 15% mass loss after 18 days at pH 5 and 30 °C and 77 days and pH 7 and 22 °C. Tao et al. coated urea granules with PLA modified with isopropyl tris(dodecylbenzenesulfonyl) titanate (TDBSP), poly(caprolactone) and acetyl tributyl citrate (ATBC) by melt blending.^[^ ^101^ ^]^ The coating allowed a N release rate of 13% after a day and a cumulative release rate of 81% after 28 days. Poly(acrylic acid) is highly hydrophilic and biocompatible, and its high content of carboxylic acid functional groups makes it pH responsive.^[^ ^108^, ^109^ ^]^ Its biodegradability depends on the molecular weight.^[^ ^110^ ^]^ Li et al. prepared a coating for ammonium dihydrogen phosphate made of poly(acrylic acid) and potassium humate, presenting interesting slow‐release and water‐preserving properties.^[^ ^102^ ^]^ The coated fertilizer released 56% phosphate and 28% ammonium in water after 28 days. Shen et al. coated urea with PAA by three different polymerization methods, achieving coatings with different hydrophobicity and glass‐transition temperatures, capable of releasing N with diverse rate patterns.^[^ ^103^ ^]^

Poly(vinyl alcohol) is a water‐soluble polymer able to form a transparent and viscous aqueous solution, ideal for forming films and membranes.^[^ ^111^ ^]^ Noppakundilograt et al. reported a trilayered coating achieved by dipping NPK granules sequentially in PVA and chitosan solutions, and then cross‐linking the chitosan layer via glutaraldehyde vapor deposition.^[^ ^104^ ^]^ The authors achieved a coating hydrogel able to delay the nutrient release over more than 30 days and efficiently absorb water (216 g/g). Nooeaid et al. incorporated NPK fertilizer in a double coating of PVA hydrophilic internal core and PLA hydrophobic external shell, to fabricate fibers with micro‐size diameter.^[^ ^105^ ^]^ The cumulative nutrient release resulted in ≈60% after 3 days, followed by a gradual increase to 80% after 28 days and a final 90% release after 90 days.

### 2.3 Biodegradable Polymers as Matrix Materials for Slow‐ and Controlled‐Release Composite Fertilizers

Slow‐ and controlled‐release fertilizers can also be prepared by incorporating the water‐soluble nutrient within an insoluble polymer matrix, able to act as a nutrient reservoir. The polymer matrix can be hydrophobic, consisting of a matrix containing the active principle, or hydrophilic, generating a hydrogel. A number of studies report the introduction of organic (e.g. cellulose nanofibers and carbon nanotubes) and inorganic (e.g. nanoclays and silica nanoparticles) fillers inside the polymer matrix, aiming at improving the fertilizer stability, mechanical properties, water retention capacity, and nutrient release kinetic. In some cases, simply blending the filler with the polymer does not allow a successful dispersion of the filler inside the matrix, due to the weak interfacial bonding between the two materials. To avoid aggregation inside the matrix, the filler surface can be modified to make it more compatible with the surrounding polymer, or polymer chains can be directly grafted onto the filler surface.

#### 2.3.1 Natural Biodegradable Polymers as Matrix for Slow‐ and Controlled‐Release Fertilizers

A wide number of studies report the incorporation of plant nutrients inside natural polymer matrix. Some examples are listed in **Table** **4**.

**Table 4:** Natural biodegradable polymers utilized as matrix in slow‐ and controlled‐release fertilizers.

Polymer	Water absorption Capacity	Nutrient	Nutrient release in water or [soil]	Ref.
Bromoacetylated cellulose	49.8 g/g	N	95.71% after 21 d	[112]
Carboxymethyl cellulose	–	N, P, K	90% in 80 h	[113]
Carboxymethyl cellulose + cellulose nanofibers	147 g/g	N	15% over 5 d, 90% over 30 d	[114]
Carboxymethyl cellulose/poly(acrylic acid)/ poly(vinylpyrrolidone) + silica nanoparticles	–	N, P, K	14.6% in 1d, 27.6% in 7 d, 54.6% in 30 d	[115]
Cellulose/poly(acrylic acid)/poly(vinyl alcohol)	243.31 g/g	N, P	50–80% in 40 min	[116]
Chitosan	3400%	Fe	50– 80% in 72 h	[117]
Chitosan/gelatin/poly(lactic acid)	4448%	N	20% in 10 min, 70% in 3 h, 86% in 15 h	[118]
Chitosan/poly(vinyl alcohol)	300%	N, P, K	50–80% in 5 d	[119]
Chitosan/poly(vinyl alcohol)	120%	N	10% in 10 d	[120]
Chitosan/Starch	160 g/g	N, K	73–95% in 14 d	[121]
Chitosan/Cellulose/poly(acrylic acid)	390 g/g	N, P, K	100% after 4.5 h [15% after 3 d, 75% after 30 d]	[122]
Chitosan + montmorillonite	140 g/g	N, P, K	[71% N, 61% P, 15% K in 12 d; 55% NPK in 15 d]	[123]
Starch	0.82 g/g	N	[95% in 15 d]	[124]
Starch	9–11%	N	[100% in 60 h]	[125]
Starch/poly(lactic acid)	–	N	100% in 28 h	[126]
Starch/poly(acrylic acid)	498 g/g	P, K	0.065% P, 70% K in 30 d	[127]
Starch/poly(acrylic acid)	700 g/g	N	25% in 5 d, >90–99% in 30 d	[128]
Starch/poly(acrylamide)	126–253 g/g	N	70% in 96 h;^[^ ^129^ ^]^ <15% in 5 d, >80% in 40 d;^[^ ^130^ ^]^ >80% in 42 h^[^ ^131^ ^]^	[129, 130, 131]
Starch carbamate/Sodium alginate	8.02 g/g	N	61.6% in 10 h, [58.5% in 25 d]	[132]
Starch/poly(β‐Cyclodextrin)/poly(acrylic acid)/poly(acrylamide) + nanotubes	71.9 g/g	N	84.6% in 12 h, [97% in 5 d]	[133]
Starch/poly(acrylic acid‐*co*‐acrylamide) + char nanoparticles	215.1 g/g	N	70% in 21 d	[134]
Starch/poly(acrylamide)/natural rubber + montmorillonite	–	N	12.70% in 1 h, 68.79% in 24 h	[135]
Sodium alginate/O‐carboxymethyl chitosan	535%	N	91% after 26 d	[136]
Sodium alginate/HTACC^a^ ^)^	300–700%	N	17–20% in 1 d, 77–85% in 30 d	[137]
Sodium alginate/poly(acrylic acid)/ poly(acrylamide) + montmorillonite	460 g/g	N, P, K	14.66% in 7 d, 57.66% in 30 d	[47]
Sodium alginate/poly(acrylic acid)/ poly(acrylamide) + attapulgite	460 g/g	N, P, K	13.44% in 1 d, 54.23% in 30 d	[138]
Sodium alginate/poly(β‐Cyclodextrin)/ poly(acrylic acid)/poly(acrylamide) + nanotubes	107.9 g/g	N	87.8% in 4 h, [79.5% in 4 d]	[139]
Sodium alginate/poly(acrylic acid‐*co*‐acrylamide) + cellulose nanocrystal	412 g/g	N	34.76% in 5 d, 86% in 15 d	[140]
Lignin	–	N	≈ 60% in 25 min	[141]
Lignosulfonate/sodium alginate	41.23 g/g	–	–	[71]
Lignin/poly(vinyl alcohol)	963.4%	P	–	[70]

^a)^ N‐(2‐hydroxy‐3‐trimethylammonium) propyl chitosan chloride.

The high density of hydroxyl groups in cellulose allows chemical modification and crosslinking.^[^ ^43^ ^]^ Mohammadi‐Khoo et al., for example, modified cellulose via bromoacetylation and then crosslinked it with urea to form a slow‐release hydrogel.^[^ ^112^ ^]^ Various cellulose derivatives were also studied as matrix for the incorporation and controlled‐release of nutrients. Carboxymethyl cellulose, for instance, has been applied for its high content of carboxylic acid groups, able to easily interact with hydrophilic and/or positively charged molecules and cations. Davidson et al. ionically crosslinked CMC chains with iron and calcium cations in presence of liquid NPK fertilizer to create a hydrogel‐based root delivery system.^[^ ^113^ ^]^ The authors managed to slow down the nutrient release over 80 h in water. Moreover, they tested the performance of the hydrogel to sustain wheat growth and observed that the lifetime of the device matches the plant lifecycle. Priya et al. synthesized hydrogels by crosslinking CMC with cellulose nanofibers and epichlorohydrin.^[^ ^114^ ^]^ This material delayed the release of urea over 30 days and lost 80% of its weight over 3 months through biodegradation. Olad et al. prepared a slow‐release fertilizer encapsulating NPK compound by in‐situ graft‐polymerization of sulfonated‐carboxymethyl cellulose with acrylic acid in the presence of poly(vinylpyrrolidone) (PVP) and silica nanoparticles as filler.^[^ ^115^ ^]^ The additive increased the hydrogel swelling capacity and acted as a crosslinking point, enhancing the network stability.

Another widely used cellulose derivative is cellulose acetate, which is less crystalline and more soluble in common solvents than cellulose. The hydroxyl groups of cellulose have also been used to graft other polymers. Li et al., for example, synthesized a semi‐interpenetrating polymer network hydrogel starting from the graft polymerization of acrylic acid on wheat straw cellulose, which is then combined with PVA to obtain a 3D structure.^[^ ^116^ ^]^ This material slowed down the release of N and P loaded in the network at different rates depending on the characteristics of the surroundings.

Chitosan is another widely explored polysaccharide to incorporate plant nutrients and slow down or control their release.^[^ ^142^ ^]^ A simple strategy for the synthesis of chitosan‐based hydrogels is through crosslinking via either chemical or physical interactions. Recently, Liu and colleagues compared the use of three different crosslinking agents (glutaraldehyde, sodium tripolyphosphate and genipin) in the development of a lightweight chitosan‐based slow‐release Fe fertilizer.^[^ ^117^ ^]^ They observed a pH‐sensitive delivery with a higher nutrient release in acidic than in neutral conditions. In addition, the genipin‐crosslinked material led to higher Fe‐loading efficiency and promoted the germination of wheat seeds and the growth of tomato plants. This highlights the importance of carefully evaluating and selecting every component of the formulation, even a small molecule as crosslinking agent, to optimize the material interaction with the plant ecosystem. Yuan et al. crosslinked chitosan with gelatin and poly(lactic acid) to encapsulate urea and control its release pattern.^[^ ^118^ ^]^ The fertilizer was delivered in water in three stages of different rates, relatively fast in the first 10 min (20% release), slower in the next 3 h (70% cumulative release) and then constant over the next 15 h (90% cumulative release). This diffusion time was 1350 times longer than the one of pure urea. Jamnongkan et al.^[^ ^119^ ^]^ and Vo et al.^[^ ^120^ ^]^ employed glutaraldehyde to crosslink chitosan and poly(vinyl alcohol) to create a blend of the two polymers, combining the biodegradability of the biopolymer and the higher hydrophilicity, mechanical properties and water retention ability of the synthetic one. In another study, Perez et al. prepared hydrogel beads via crosslinking chitosan and starch with sodium tripolyphosphate, showing the influence of crosslinking time and starch incorporation on the physical properties of the final product as well as on the nutrient release kinetics.^[^ ^121^ ^]^ Essawy et al. prepared a superabsorbent hydrogel characterized by a water absorption capacity in the order of several hundred times their dry weight.^[^ ^122^ ^]^ They first hybridized chitosan with cellulose to improve the mechanical strength of the chitinous material. Then, the amine and hydroxyl groups of the product were used as anchor sites for graft polymerization of acrylic acid. The final material showed mechanical robustness, pH‐responsive behavior and slower nutrient release compared to free NPK in water. Dou et al. fabricated a highly swellable and biodegradable hydrogel by crosslinking chitosan with montmorillonite nanoclays, in which NPK fertilizer was incorporated.^[^ ^123^ ^]^ Interestingly, an increasing content of this filler reduced the N release rate, and slightly accelerated the delivery of P and K.

Starch is another polysaccharide widely applied to contain and deliver nutrients. The presence of many hydroxyl groups allows the interaction with water and nutrient molecules, promoting good water absorption and holding capacity that facilitate the production of starch‐based superabsorbent hydrogels. Hydroxyl groups also help the modification of starch by introducing new moieties or inducing covalent crosslinking through different methodologies. Various scientific articles describe the fabrication of starch hydrogels for controlling N release, where urea is utilized both as active ingredient and as a plasticizer. An example was published by Versino et al., who prepared starch films containing 0–50%wt. of urea.^[^ ^124^ ^]^ Urea resulted in being an efficient plasticizer, making the films more flexible and resistant, and increasing the material biodegradation in soil. The films with 50%wt. urea released less than 95% fertilizer within 15 days in soil experiments. Rychter et al. have extruded starch films made of 25%wt. glycerol as plasticizer and containing up to 10%wt. urea.^[^ ^125^ ^]^ Films with the highest urea concentration provided the most prolonged release (100% after 60 h), were non‐toxic, and able to stimulate the growth of both *Avena Sativa* and *Raphanus sativus* in pot experiments. Starch presents poor mechanical resistance and plasticity,^[^ ^35^ ^]^ and is often blended with synthetic polymers to improve the mechanical performance and at the same time maintain a good level of biodegradability of the final material. Chen et al. encapsulated urea into a film consisting of starch modified with polylactic acid by in situ graft‐copolymerization of lactide.^[^ ^126^ ^]^ The addition of hydrophobic poly(lactic acid) could reduce swelling of the starch matrix, reducing the urea release rate. The release properties could be modified by adjusting the graft efficiency, which allowed to tune the nutrient delivery from several hours to one day. Zhong et al. embedded phosphate rock in a superabsorbent polymer made of sulfonated corn starch and poly(acrylic acid).^[^ ^127^ ^]^ The final material showed great swelling capacity, up to 498 g/g, and a sustained release of P and K. Starch was also combined with polyacrylic acid by Sarmah et al., who developed a superabsorbent hydrogel with 700 g/g water absorption capacity.^[^ ^128^ ^]^ The authors encapsulated urea into the hydrogel and tested the slow‐release fertilizer on *Cicer arietinum* plant, achieving a release over 30 days. Poly(acrylamide) grafted starch‐based hydrogels have been synthesized for urea delivery employing different synthetic technologies, such as a one‐step process of reactive melt mixing,^[^ ^129^ ^]^ reactive extrusion^[^ ^130^ ^]^ and *in situ* radiation‐synthesis.^[^ ^131^ ^]^ The results showed the importance of the synthetic process on the final properties of the slow‐release hydrogel, as well as the urea incorporation method (e.g., encapsulation, matrix dispersion) and the starch botanical origin. The source of starch determines the ratio between α‐amylose and amylopectin, which has a deep influence on the intra‐ and inter‐molecular forces and the starch chemical reactivity.^[^ ^35^, ^129^ ^]^ In another example, a biopolymer‐based starch carbamate and sodium alginate hydrogel was synthesized via cationic and hydrogen bond crosslinking for urea delivery. The product succeeded in reducing the nutrient delivery rate and promoted the growth of maize seedlings in pot experiments.^[^ ^132^ ^]^ In this study, the water absorbency level reached 8.02 g/g and urea was completely released in water in 16 h, while the enrichment of starch hydrogels with polymers like poly(acrylic acid) or poly(acrylamide) led to swelling capacities of hundreds of times their dry weight and delays in nutrient delivery in water up to 30–40 days. Wei et al. fabricated a complex system via free radical copolymerization of starch, acrylic acid, acrylamide and β‐cyclodextrin, in which halloysite nanotubes loaded with urea were encapsulated. The addition of nanotubes reduced the release rate of urea, and the slow‐release fertilizer was able to absorb water and increase the water retention behavior of a sandy soil in which the samples were tested.^[^ ^133^ ^]^ Salimi et al. reported the reinforcement of starch‐*g*‐poly(acrylic acid‐co‐acrylamide) hydrogel used for encapsulation and slow delivery of urea with natural char nanoparticles.^[^ ^134^ ^]^ An increase in the nanoparticle content in the system resulted in prolonging the fertilizer releasing time. This was attributed to the efficient interaction between polymer and filler, able to slow down the N diffusion through the matrix. The additive also allowed to double the water retention of the soils in which they were tested, as compared with the neat polymer. Jumpapaeng et al. fabricated an urea slow‐release hydrogel via free‐radical polymerization of starch, poly(acrylamide), natural rubber, and montmorillonite (0–10 wt.% content).^[^ ^135^ ^]^ An increase in the filler content corresponded to slowing down the nutrient release. Moreover, the composite containing 3 wt.% of filler resulted in increasing the mechanical strength by 260% and the biodegradation rate of 58% compared with the control without montmorillonite.

Alginate contains carboxylic acid and hydroxyl groups that facilitate chemical modifications, crosslinking with multivalent cations and grafting of polymers. The carboxylic acid moieties make this polymer, and the materials based on it, pH sensitive.^[^ ^143^, ^144^ ^]^ Arafa et al. crosslinked sodium alginate with O‐carboxymethyl chitosan using calcium chloride as a crosslinker.^[^ ^136^ ^]^ The obtained hydrogels were able to slowly release urea and showed interesting antimicrobial properties, thanks to the presence of chitosan. In another study, the same authors developed a superabsorbent hydrogel for the incorporation and slow release of urea made of sodium alginate crosslinked with N‐(2‐hydroxy‐3‐trimethyl ammonium) propyl chitosan chloride.^[^ ^137^ ^]^ A variation in the content of the two components, with alginate increased from 50% to 70%, led to an increase in both the swelling degree and urea loading in the samples, reaching a maximum of 200% loading. Rashidzadeh et al. tested the loading of two different clay materials, namely montmorillonite and attapulgite, in a sodium alginate/poly(acrylic acid)/poly(acrylamide) matrix, successfully decreasing the NPK release rate.^[^ ^47^, ^138^ ^]^ In another study, Shen et al. exploited the hollow structure and large surface area of halloysite nanotubes to load urea and subsequently incorporated them in sodium alginate‐based double‐network hydrogel particles.^[^ ^139^ ^]^ Idrissi et al. demonstrated the advantage of incorporating organic fillers, such as citric acid‐grafted cellulose nanocrystals in a sodium alginate‐based hydrogel.^[^ ^140^ ^]^ The additives were used both as physical crosslinkers and to improve the swelling capacity and the slow‐release properties of the material. Plant experiments demonstrated their potential to promote tomato growth under water stress.

Lignin has also been widely used for the encapsulation of urea. Mulder et al. have selected a commercial lignin that was able to incorporate urea inside a film and to delay its release to ≈60% in 25 min.^[^ ^141^ ^]^ This result was achieved by adding alkenyl succinic anhydride to the lignin, which increased the hydrophobicity of the coating and hence reduced its water‐sensitivity. Song et al. have crosslinked lignin with sodium alginate and tested its ability to reduce the leaching of N, P, and K from the polymer matrix.^[^ ^71^ ^]^ The application of the product on tobacco plants significantly increased its mass harvest. Khan et al. crosslinked kraft lignin with poly(vinyl alcohol) employing epichlorohydrin in the presence of struvite, a mineral that can be precipitated from waste sludge and represent a renewable source of P.^[^ ^70^ ^]^ A sustained P release over 6–8 h was observed, combined with good water absorption capacity, showing the potential of lignin‐based hydrogel in the slow delivery of fertilizers.

#### 2.3.2 Synthetic Biodegradable Polymers as Matrix for Slow‐ and Controlled‐Release Fertilizers

The use of synthetic biodegradable polymers for the production of films and hydrogels for agricultural applications is relatively limited, as summarized in **Table** **5**.

**Table 5:** Examples of synthetic biodegradable polymers used as matrix in slow‐ and controlled‐release fertilizers.

Polymer	Water absorption capacity	Nutrient	Nutrient release in water or [soil]	Ref.
Poly(lactic acid)	–	N	75% in 168 h	[145]
Poly(vinyl alcohol)	–	P	0.5% in 30 d	[146]
Poly(vinyl alcohol)/poly(ethylene glycol)	250%	N	20–30% in 1 d	[147]
Poly(vinyl alcohol)/soy protein isolate	>200%	N	87.5% in 10 d, [14.3% in 1 d, 74.1% in 28 d]	[148]
Poly(vinyl alcohol) + montmorillonite	≈ 250%	K, P	10% in 1 d, 50–60% in 19 d	[149]
Poly(vinyl alcohol) + kaolin	66.6 g/g	N, P	88.8% P in 30 d, [76.3% P in 30 d]	[150]
Poly(acrylic acid)	1000 g/g	N, P, K	≈20% in 1 d, 90–100% in 15 d	[151]
Poly(acrylic acid)	>150 g/g	K, P	–	[152]
Poly(acrylic acid)	909 g/g	N	0.18% in 1 d, 2.67% in 30 d	[153]
Poly(acrylic acid) + cellulose nanofibers	≈ 300 g/g	N	–	[154]

Kaavessina et al. incorporated urea in low molecular weight poly(lactic acid) to formulate slow‐release fertilizer, achieving a nutrient delivery of 75% in 168 h.^[^ ^145^ ^]^ The delayed release of urea was attributed to a combination of fertilizer diffusion through the matrix and PLA degradation. A controlled‐release fertilizer was prepared by Zhang et al., who encapsulated ferric phosphate in a poly(vinyl alcohol) film.^[^ ^146^ ^]^ The PVA matrix allowed a tenfold faster release of phosphate in the presence of citric acid, which mimics the rhizosphere around plant roots, as compared to pure water. The matrix showed water retention for up to 30 days and a nutrient release for longer than 28 days, promising results for the application as slow‐release fertilizer. Sarkar et al. crosslinked PVA with poly(ethylene glycol) to create a hydrogel with a dual function: on the one hand urea loading and slow delivery, and on the other hand adsorption of Fe (III) from soil, aiming to reduce iron toxicity in rice plants.^[^ ^147^ ^]^ In another study, citric acid was used to crosslink poly(vinyl alcohol) and soy‐protein isolate to form a hydrogel that was loaded with urea.^[^ ^148^ ^]^ Hakim and colleagues decreased the release of potassium phosphate by crosslinking PVA with glutaraldehyde in the presence of montmorillonite.^[^ ^149^ ^]^ The results showed that the higher the concentration of the phyllosilicate, the more delayed the nutrient delivery at the cost of a lower water absorption capacity. Sharma et al. prepared a PVA hydrogel incorporating kaolinite as mechanical binder and diammonium hydrogen phosphate as fertilizer.^[^ ^150^ ^]^ The addition of the filler reduced the swelling ratio of the hydrogel, allowing to tune the nutrient release rate from the matrix.

Many of the commercially available hydrogel products for agricultural applications are based on poly(acrylic acid).^[^ ^155^ ^]^ Previously, the focus was on optimizing the parameters for the development of pure synthetic hydrogel materials. For instance, Teodorescu et al. incorporated NPK liquid fertilizers in crosslinked PAA hydrogels, which displayed good nutrient release properties and excellent swelling ratios, with a water uptake of up to 1000 g/g.^[^ ^151^ ^]^ In a similar study, Tyliszczak et al. synthesized a PAA hydrogel in the presence of granular phosphorous fertilizer KH_2_PO_4_.^[^ ^152^ ^]^ Cheng et al exploited the chemical structure and reactivity of urea and embedded it in the hydrogel matrix, using it as a crosslinker. Their strategy demonstrated good efficacy for the production of a hydrogel with very long time delivery, considering that only 2.7% of the urea was released in water after 30 days.^[^ ^153^ ^]^ Nowadays, most of the investigations on PAA in agriculture see it combined with other materials more prone to biodegradation to optimize the environmental compatibility of these products at different time scales. Shahzamani et al. incorporated different quantities of urea in a film of PAA and cellulose nanofibers.^[^ ^154^ ^]^ The authors found out that a content of 5wt% filler and a ratio 3:10 urea:polymer was the ideal formulation to achieve maximum water absorbency and mechanical reinforcement from the cellulose nanofibers.

### 2.4 Nanofertilizers

Nanotechnology has been successfully incorporated into the concept of slow‐ and controlled‐release fertilizers. The high surface‐to‐volume ratio, reduced size and control of surface functionalization allow to tune the properties of release, increase shelf‐life, and prevent premature degradation and loss of active ingredients, thereby contributing to improved resource use efficiency and more sustainable agricultural practices.^[^ ^7^, ^10^, ^156^, ^157^, ^158^, ^159^ ^]^ Nanofertilizers are generally considered materials characterized by sizes that range from 1–100 nm,^[^ ^160^ ^]^ even though in many reported studies this range is extended up to 1000 nm. The small size promotes the interaction with the plant and can enhance the uptake by the leaves and roots.^[^ ^159^ ^]^ However, this property needs to be tuned considering the different barriers that the nanocarriers have to overcome on the leaf and root surfaces and within the plant. In addition, the permeation success within the plant depends not only on the size, but on various other parameters such as the aspect ratio, the surface charge and chemistry, and the stiffness.^[^ ^157^, ^159^ ^]^ At nanoscale dimensions, surface effects have great importance. This causes strong interactions between plants and nanomaterials, which can induce specific responses by the plant (e.g., modification of reactive oxygen species and antioxidative enzyme activity levels).^[^ ^158^ ^]^ Thus, nanofertilizers can promote plant growth not solely based on the slow delivery rate of nutrients, but also by reinforcing its development under biotic and abiotic stresses by finely tuning the nanocarrier design choosing the appropriate material, surface chemistry, and dimensions. To minimize nutrient losses and match the release rates of nutrients with the crop needs, there is increasing interest to develop nano‐sized formulations that can actively respond to changes in environmental and biochemical parameters (e.g., pH, temperature, enzymatic activity).^[^ ^161^ ^]^

Among polymeric materials used to build nanofertilizers, most of the research focused on bio‐derived polymers, especially chitosan.^[^ ^162^, ^163^ ^]^ Concerning other natural polymers, such as cellulose,^[^ ^41^, ^164^ ^]^ lignin,^[^ ^69^, ^165^ ^]^ and alginate,^[^ ^160^ ^]^ some studies have been conducted, as reported in **Table** **6**, but there is ample opportunities to explore the potential of biodegradable polymers for the development of nanofertilizers.

**Table 6:** Biodegradable polymers utilized in nanofertilizer formulation. The nanofertilizer diameter is reported as analyzed with transmission electron microscopy (TEM), scanning electron microscopy (SEM), and dynamic light scattering (DLS).

Polymer	Diameter [nm]	Zeta potential [mV]	Nutrient	Application type/Plant	Ref.
Cellulose nanofibers	–	–	K	Foliar/Onion	[166]
Cellulose nanofibers	SEM: 17.02 ± 4	−42	N	–	[167]
Chitosan/poly(methacrylic acid)	TEM: 78 DLS: 140–220	+ 10–20	N, P, K	–	[168]
Chitosan/poly(methacrylic acid)	TEM: 17–25	+ 33.6–85.4	N, P, K	Foliar/Wheat	[169, 170, 171]
Chitosan/sodium tripolyphosphate	SEM: 200 DLS: 290–325	+ 32.2–42	Zn	Foliar/Durum Wheat	[172]
Chitosan/sodium tripolyphosphate	TEM: 500	+ 50	N, P, K	Foliar/Coffee	[173]
Chitosan/poly(methacrylic acid)	TEM: 39–79 SEM: 90 ± 65 DLS: ≈100	+ 15.6–21.8	K	Soil/Maize	[174]
Chitosan/sodium tripolyphosphate	DLS: 360.5 ± 1.3	+ 37.1 ± 0.32	Si	Seed and Foliar/Maize	[175]
Chitosan/starch/zein/cellulose acetate/poly(caprolactone)	SEM: 159–170	–	N, P, K, S	Soil/Soybean – Wheat	[176]
Chitosan/sodium tripolyphosphate	DLS: 301 – 430	+ 5.8–13.8	P, Zn	Soil/Wheat	[177]
Chitosan/poly(acrylic acid)	SEM: 46–200	–	N	Soil/Corn	[178]
Sodium alginate	TEM: 100–300 DLS: 287–331	−19.6–(−22.8)	Fe	–	[179]
Sodium alginate/chitosan	SEM: > 150 DLS: ≈ 100–600	–	K	–	[180]
Sodium alginate/chitosan	DLS: 316.3–450.9	−24–(−33)	N, P, K	–	[181]
Aminated lignin	DLS: 100–800	+ 9.59–27.8	P	–	[182]

Due to its insolubility, the application of pure cellulose can be challenging. Different chemical and mechanical processes can improve its properties. Cellulose nanofibrils are elongated cellulosic structures with a nanosized diameter (3–10 nm), and lengths in the micrometer range. They have several interesting properties, such as a high aspect ratio, large specific surface area, and good mechanical strength. They are produced starting from cellulose fibers, which undergo chemical modifications (e.g., TEMPO oxidation) followed by mechanical processes. They have been used in several applications in agriculture mainly as coating materials or as components in composite delivery products.^[^ ^164^ ^]^ A few examples showed the potential of these structures to be used directly as a controlled‐release system. Abd El‐Aziz et al. produced TEMPO‐oxidized cellulose nanofibers (TO‐CNFs) in the presence of potassium hydroxide to produce potassium carboxylate TO‐CNFs and grafted potassium poly(acrylate) onto TO‐CNFs as K sources for the plant.^[^ ^166^ ^]^ Field experiments showed that foliar‐applied potassium carboxylate‐modified nanofibrils increased the growth of onion plants by up to 50% for two growing seasons. In another study, researchers focused on investigating the biodegradability of N‐loaded cellulose nanofibers and the effect of the degradation process on the soil environment and its microbiome.^[^ ^167^ ^]^ The results were promising, making them interesting candidates for field trials, as 62% of the material was degraded after 36 days, microbial differentiation was preserved and its growth was promoted.

Chitosan‐based nanoparticles are among the most studied systems for the development of nanofertilizers. These systems stimulate plant growth and development, enhance plant protection against biotic and abiotic stresses, and improve soil quality.^[^ ^162^, ^163^ ^]^ The presence of amine groups provides chitosan with a pH‐dependent solubility and a cationic nature at pH < 6–6.5. Thus, chitosan nanomaterials' positive zeta potential favors their interaction with biological systems, as they are attracted by the net negative surface charge of most cell membranes. This polymer also provides antimicrobial ability, and can facilitate penetration within the plant, which can enhance the overall payload delivery efficiency. In most reported studies, chitosan nanofertilizers were produced via simple and cost effective methods, such as ionotropic gelation, by dropwise addition of an anionic crosslinker, like sodium tripolyphosphate (TPP), to a chitosan solution and polyelectrolyte complexation of chitosan chains with e.g., poly(methacrylic acid) (PMAA).^[^ ^162^, ^163^ ^]^ Corradini et al. synthesized chitosan‐PMAA chitosan nanoparticles obtained by polymerization of methacrylic acid in a chitosan solution and loaded NPK nutrients in a second step.^[^ ^168^ ^]^ Other researchers developed a similar nanoplatform based on chitosan and PMAA, achieving a smaller size (17–25 nm average diameter from TEM) and higher surface charge, with values up to 85.4 mV.^[^ ^171^ ^]^ These nanofertlizers were applied via foliar spraying in field studies on wheat.^[^ ^169^, ^170^ ^]^ These experiments revealed the presence of nanoparticles in the phloem tissue, demonstrating their uptake through the stomata and translocation within the plant. Plant growth and productivity were accelerated under nanofertilizer regime compared to conventional fertilizer. The authors also observed an increase in wheat grain quality. However, the plant response to this new fertilizer varied based on several factors, like soil type, plant species, formulation type, and concentration. Kubavat et al. developed K‐loaded chitosan/PMAA nanoparticles intending to understand the nutrient delivery kinetics in water and soil, the nanofertilizer/soil interaction and the nanofertilizer effect on plants via soil application.^[^ ^174^ ^]^ The nanoformulation provided a sustained release of potassium and led to a higher biomass production when applied in maize in pot experiments at a reduced potassium rate (75% nanofertilizer) compared to the positive control (100% KCl). In addition, the nanoformulation modified soil characteristics in terms of increased porosity and lower bulk density. Concerning chitosan nanoparticles crosslinked with TPP, the crosslinking agent is readily prone to hydrolysis in solution and a source of P nutrient. This system is characterized by a facile encapsulation of different chemicals, and it has been employed for the delivery of various nutrients. Deshpande et al. developed chitosan/TPP nanocarriers loaded with zinc, a fundamental micronutrient for plant growth, thanks to the chelating abilities of chitosan and the anionic nature of TPP.^[^ ^172^ ^]^ Durum wheat plants foliar treated with these nanoparticles showed increased Zn seed content, demonstrating the efficient delivery. Ha et al. loaded NPK fertilizer within chitosan/TPP nanoparticles and tested their effect on coffee seedlings in greenhouse studies.^[^ ^173^ ^]^ The plants treated with the nanofertilizer demonstrated increased growth levels in terms of plant height, leaf number and area as well as NPK leaf content and photosynthesis net rate. In a more recent study, Dimpka et al. functionalized chitosan/TPP nanoparticles with ZnO nanofiller and studied the chitosan‐mediated delivery and uptake of P and Zn by the plant.^[^ ^177^ ^]^ In addition, this study showed the use of TPP as a P source for the plant and the reduction of P loss through leaching that can be obtained by incorporating the nutrient in the nanoparticle matrix as the crosslinking agent. Other synthetic pathways for the synthesis of efficient chitosan‐based nanofertilizers have been studied. Xu et al., for instance, employed co‐axial electrospray to synthesize core‐shell nanoparticles made of different polymeric blends mixing to different extents: chitosan, starch, zein, cellulose acetate and poly(caprolactone).^[^ ^176^ ^]^ Due to the nature of the materials employed, the nanocarriers demonstrated a pH‐ and enzyme‐sensitive delivery of the incorporated nutrients (CuSO_4_ and NPK). Piroonpan and colleagues, recently, developed an ammonium nitrate‐loaded pH‐responsive system based on copolymerized chitosan/PAA nanoparticles employing energetic radiation‐induced free radical graft copolymerization.^[^ ^178^ ^]^ The N release profiles at different pH levels changed based on the swelling behaviors. The nanoformulation enhanced plant growth even in sandy soil compared to the control and demonstrated its properties as a soil conditioner.

Another biopolymer prone to facile nanoparticle synthesis via ionic crosslinking is sodium alginate, thanks to its carboxylic acid functional groups. A typical crosslinking agent is calcium in its cationic form. Patel et al. investigated the swelling behavior of Ca‐crosslinked alginate nanoparticles in dependence on the extent of the calcium content and crosslinking of the biopolymer.^[^ ^179^ ^]^ They also added iron to the formulation, as a plant micronutrient, and observed the direct correlation between the swelling extent and the delivery rate of Fe ions. Another common strategy to produce alginate‐based nanoparticles is by complexing the negatively charged algae‐derived biopolymer with a positively charged polymer, like chitosan. Hamed et al. developed a novel nano bio‐fertilizer utilizing these two natural polymers.^[^ ^181^ ^]^ Humic acid was added to improve the crosslinking, the water‐holding capacity and the encapsulation efficiency. The capsules were loaded with NPK fertilizer and growth‐beneficial bacteria, which are microorganisms that once released close to the plant, colonize the rhizosphere and promote plant growth and development.

The use of lignin‐based nanoparticles in agriculture has been mainly focused on the delivery of pesticides, fungicides and insecticides, exploiting the intrinsic properties of lignin to e.g., elongate the lifetime of active ingredients providing UV protection, and its sensitivity toward enzymes produced by pests and microbes, like laccases.^[^ ^161^, ^165^ ^]^ One example of the use of lignin for the development of slow‐ and controlled‐delivery nanofertilizers, is a study by Li et al, who developed lignin‐based magnetic nanoparticles for nutrient recovery from wastewater and their reutilization as slow‐release fertilizers.^[^ ^182^ ^]^ Fe_3_O_4_ nanoparticles and Fe^3+^ were incorporated in aminated lignin nanocarriers and their P adsorption properties were investigated. Moreover, the ability of the system to slowly release Fe and P in water was demonstrated.

## 3 Biodegradable Polymers for Nutrient Recovery

The recovery of nutrients from wastewater and other waste streams plays a key role in advancing sustainability and reducing environmental pollution. This is particularly crucial for N and P. While fertilizers based on these two elements are essential for agriculture, their production is energy intensive and accompanied by negative environmental consequences. Moreover, their run off and leaching to waterbodies leads to eutrophication and excessive algae growth.^[^ ^12^, ^183^, ^184^, ^185^ ^]^ 20–70% of N applied to soil is lost in the environment via NH_3_, N_2_O, and NO volatilization and NO_3_^−^ leaching in the groundwater.^[^ ^183^, ^186^ ^]^ The efficiency of P fertilizers is also very low, as only 15–25% of the P applied to soil is absorbed by crops, while the rest is fixed in soil in unavailable forms or lost via leaching to the groundwater and via runoff to surface water bodies.^[^ ^185^, ^187^, ^188^ ^]^

Technologies for the recovery of N and P from wastewater not only have central importance in reducing environmental impact, but also offer opportunities for the production of high‐value products, such as green fuels and fertilizers. One efficient method for nutrient recovery is the precipitation of struvite (NH_4_MgPO_4_·6H_2_O)^[^ ^189^, ^190^, ^191^, ^192^ ^]^ or hydroxyapatite (Ca_5_(PO_4_)_3_OH),^[^ ^193^, ^194^ ^]^ which allow to remove up to 80–90% of N and P from wastewater and generate minerals that can be commercialized as slow‐release fertilizers.^[^ ^195^, ^196^ ^]^ The efficiency of these precipitation processes is limited by the concentration of P in wastewater. If this is low, the process can have significantly lower yields or require longer reaction time, influencing the economic applicability of the precipitation.^[^ ^15^ ^]^ Another approach is ammonia stripping, which allows to recover up to 92% of N from wastewater, but its limitations are the high energy demand and the use of hazardous reagents that increase the process costs and complicate the maintenance of the plants.^[^ ^197^, ^198^, ^199^ ^]^ The use of biodegradable polymers provides alternative, safe and cost‐efficient methods for the recovery of nutrients from wastewater.

The following sections will highlight membrane filtration, adsorption and coagulation/flocculation; three methods for nutrient recovery where biodegradable polymers offer significant opportunities.

### 3.1 Biodegradable Polymer Membranes for Wastewater Treatment

Membrane separation includes a wide category of techniques for nutrient recovery, which does not require additional reagents nor produces unwanted by‐products.^[^ ^14^, ^15^, ^200^ ^]^ Membranes can be generally made of polymeric, ceramic and biological materials. Polymeric membranes are the most widely utilized for water treatment, thanks to their thermal, mechanical and chemical properties.^[^ ^200^, ^201^ ^]^ Membranes are selective barriers able to regulate the transport of substances between two phases.^[^ ^201^, ^202^ ^]^ In the case of wastewater purification, when the feed solution containing nutrients and contaminants crosses the membrane these impurities are retained, while purified water can pass. This process allows to recover a solution rich in nutrients, which can be used for crop irrigation. This practice, called fertigation, aims at recycling nutrients and lowering the use of additional mineral fertilizers.^[^ ^203^, ^204^ ^]^ Polymers allow the preparation of membranes with attractive mechanical properties, robustness across a wide range of pH, isoporosity, and high pore density.^[^ ^200^, ^202^, ^205^, ^206^, ^207^ ^]^ Polymer membranes applied for wastewater treatment are typically prepared via phase inversion.^[^ ^207^, ^208^ ^]^ The majority of the polymers used in the fabrication of these membranes are currently fossil‐based like poly(vinylidene fluoride),^[^ ^209^, ^210^ ^]^ poly(propylene),^[^ ^211^ ^]^ poly(tetrafluoroethylene),^[^ ^212^ ^]^ poly(sulfone),^[^ ^213^ ^]^ poly(acrylonitrile)^[^ ^214^ ^]^ and poly(amide).^[^ ^215^, ^216^ ^]^ The substitution of these polymers with biodegradable ones is relevant to reduce the environmental impact of this widely utilized technology. **Table** **7** lists several examples of membranes made of biodegradable polymers that have been used for nutrient recovery.

**Table 7:** Biodegradable polymers utilized in membranes for nutrient recovery (N. S. stands for non‐specified).

Polymer	Recovery method	Preparation	Recovered Nutrient	Recovery [%]	Ref.
Cellulose acetate	Reverse osmosis	N. S.	N	98	[217]
Cellulose acetate	Reverse osmosis	N. S.	N	99	[218]
Cellulose acetate	Reverse osmosis	Phase inversion	K	–	[219]
Poly(lactic acid)	Ultrafiltration	Phase inversion	N, P	96 N, 52 P	[220]
Poly(lactic acid)	Nanofiltration	Phase inversion	N, P	90 N, 71 P	[221]
Poly(vinyl alcohol)/cellulose ester	Forward osmosis	Phase inversion + crosslinking	N	–	[222]

Membranes made of cellulose acetate have been widely reported for their high hydrophobicity, biocompatibility, toughness and antifouling properties.^[^ ^223^ ^]^ Bódalo et al. demonstrated the efficiency of commercially available cellulose acetate membranes for reverse osmosis in recovering ammonium from aqueous solutions.^[^ ^217^ ^]^ More than 98% of ammonium was retained by the membrane in all the tested conditions. Beside the excellent filtration efficiency of cellulose acetate, it can be necessary to improve its properties in case of harsh conditions or high pressure.^[^ ^223^ ^]^ An example of this case was reported by Carter et al., who compared the efficiency of two commercially available membranes, one made of poly(amide) and another one of cellulose acetate, to retain ammonium sulfate from digestate, the liquid by‐product of anaerobic digestion, a process converting biomass waste into biogas.^[^ ^218^ ^]^ Both membranes were able to retain at least 99% of ammonium at the pressures specified by the manufacturer. However, when the authors tested the membranes with a higher pressure than the one recommended by the supplier, the compaction effects resulted reversible only for the membrane made of poly(amide), not for the one in cellulose acetate. A more complex system was developed by Casadellà et al., who prepared a polymer inclusion membrane (a particular type of membrane well‐described by Nghiem et al.^[^ ^224^ ^]^) using cellulose triacetate as the main matrix, 2‐nitrophenyloctyl ether as plasticizer and dicyclohexan‐18‐crown‐6 as carrier.^[^ ^219^ ^]^ This membrane was able to selectively retain K^+^ from urine over competitive ions such as Na^+^ and NH_4_^+^. Nassar et al. have published two studies about poly(lactic acid) membranes for ammonium and phosphate recovery. In one case, ultrafiltration membranes were synthesized by non‐solvent induced phase inversion method combining poly(lactic acid), N‐dimethylacetamide and poly(vinylpyrrolidone) in different ratios.^[^ ^220^ ^]^ The best filtration performance was achieved by membranes with the highest content of PLA (20%wt.), capable of retaining 96% and 87% of NH_4_^+^, from synthetic and raw wastewater, respectively, which are better results compared to other commercially available membranes made of conventional oil‐derived polymers. The high efficiency in ammonium removal was attributed to a combination of adsorption and electrostatic repulsion. This membrane could also efficiently retain 22% and 52% phosphate from synthetic and raw wastewater, respectively, mainly thanks to electrostatic repulsion forces. Due to the hydrophobic nature of PLA, however, increasing its content led to a reduction in the water permeability of the membrane. The authors have hence improved this first PLA membrane by incorporating positively charged multi‐walled carbon nanotube and negatively charged graphene oxide, aiming to target the retention of the oppositely charged NH_4_^+^ and phosphate while enhancing the water permeability.^[^ ^221^ ^]^ The incorporation of only 1.5 wt.% of the additives into the PLA matrix allowed to increase the water flux of 74% compared to the unmodified membrane, and to retain 90% ammonium and 71% phosphate from raw wastewater. Gonzales et al. have covered cellulose ester membranes for forward osmosis with a PVA hydrogel prepared by crosslinking with glutaraldehyde and borax.^[^ ^222^ ^]^ This composite membrane showed high ammonium retention and the hydrophilic PVA layer increased the resistance to fouling.

### 3.2 Biodegradable Polymer‐Based Adsorbents

Adsorption is another widely used technique, based on the separation of nutrients from wastewater by binding them on an adsorbent material such as polymer, graphene oxide, biochar, and zeolite.^[^ ^225^, ^226^, ^227^ ^]^ The adsorption mechanism can be based on electrostatic interactions, ion exchange, ligand exchange, precipitation or complexation between the nutrients and the adsorbent.^[^ ^225^, ^226^, ^227^ ^]^ Adsorption for the treatment of wastewaters can be performed in batch reactors or continuously in fixed‐bed reactors or columns. Although this process is efficient in removing nutrients from the substrate, the final step of regeneration is quite expensive and hinders a wider application of this method.^[^ ^228^, ^229^ ^]^ This process involves transferring pollutants and nutrients from a liquid phase to a solid adsorbent. The performance of an adsorbent material can be assessed by the adsorption capacity, which measures the amount of nutrient adsorbed per unit mass of the adsorbent material. It is determined by subtracting the final nutrient concentration from the initial concentration, then dividing this value by the mass of the adsorbent material and multiplying by the volume of the solution. This provides the amount of nutrient adsorbed in milligrams per gram of adsorbent.^[^ ^230^ ^]^

After use, adsorbents can be recovered and regenerated.^[^ ^231^ ^]^ This cycle can often be repeated multiple times, but regenerated adsorbents typically exhibit lower adsorption capacity compared to their pristine counterparts. Regeneration methods include magnetic separation, filtration, thermal desorption, solvent regeneration, microwave irradiation, supercritical fluid regeneration, advanced oxidation processes, and microbial‐assisted regeneration.^[^ ^231^ ^]^ Each method has its own advantages and disadvantages, so it is always important to evaluate the best regeneration strategy for reusing spent adsorbents. A range of adsorbent materials has been utilized for nutrient removal, including zeolites,^[^ ^232^ ^]^ activated carbon,^[^ ^233^ ^]^ graphene oxide,^[^ ^234^, ^235^ ^]^ metal‐organic frameworks,^[^ ^236^ ^]^ biochar,^[^ ^237^ ^]^ and polymer‐based composites.^[^ ^226^, ^227^, ^238^ ^]^ Polymer‐based adsorbents have recently emerged as highly effective options for the decontamination of polluted water and nutrient recovery, thanks to their great adsorption capacity and biocompatibility. Polymer‐based adsorbents can be used alone, employing the interaction between the polymer functional groups and the targeted nutrient, or in combination with different fillers. Among the fillers utilized with this aim, Fe_3_O_4_ can be used to promote the interaction with phosphate in a pH range between 2 and 7.^[^ ^239^ ^]^ Various La^3+^‐based compounds can also be immobilized in a polymer matrix to increase the adsorption of phosphate and nitrate, forming the stable salts LaPO_4_ and La(NO_3_)_3_.^[^ ^240^, ^241^ ^]^ **Table** **8** highlights several examples of biodegradable polymers that have been used as adsorbent composites for nutrient recovery.

**Table 8:** Biodegradable polymers utilized as adsorbent for nutrient recovery.

Polymer	Recovery method	Preparation	Recovered nutrient	Adsorption capacity [mg g^−1^]	Ref.
Chitosan	Nanoparticles	–	N	0.769	[242]
Chitosan	Beads	Crosslink	N, P	47 NO_3_^−^, 43 NO_2_^−^, 117 P	[243]
Chitosan	Beads	Crosslink	P	52.1	[244]
Chitosan/biochar	Film	Precipitation	N, P	3.5 N, 7.5 P	[245]
Chitosan/bentonite	Beads	Coagulation	N	11.6	[246]
Chitosan/banana peel	Powder	Grafting	N, P	42.16 N, 15.91 P	[247]
Chitosan/LaCl_3_	Membrane	Casting method	N, P	62.6 N, 76.6 P	[248]
Lignin	Powder	Amination	P	46.28	[249]
Lignin/Fe_3_O_4_	Nanoparticles	Grafting + precipitation	P	–	[182]
Lignin/Fe_3_O_4_	Nanoparticles	Amination + precipitation	P	43	[250]
Lignin/LaN_3_O_9_	Powder	Precipitation	P	107	[251]
Lignin/LaCl_3_	Nanoparticles	Grafting + precipitation	P	65.79	[252]
Calcium alginate	Beads	Crosslink	N, P	4.75 N, 1.4 P	[230]
Sodium alginate/bentonite/hydrotalcite/metal ions	Beads	Crosslink	P	30.25	[253]
Sodium alginate/Fe_3_O_4_	Beads	Precipitation	N, P	23.83 N, 30.14 P	[239]
Sodium alginate/ graphene oxide	Beads	Coagulation	N, P	51.83 N, 58.46 P	[254]
Poly(vinyl alcohol)	Film	Crosslink	P	28.15	[255]
Poly(vinyl alcohol)	Film	Crosslink	P	99	[256]
Poly(acrylic acid)	Hydrogel	Polymerization	N	8.8	[257]
Poly(acrylic acid)/sodium alginate/TiO_2_	Hydrogel	Polymerization	N	112.9	[258]

Chitosan is a biocompatible and low‐cost adsorbent, able to interact with nutrients and contaminants via its amino and hydroxyl groups.^[^ ^227^ ^]^ The main mechanism of NO_3_^−^, NO_2_^−^, and HPO_4_^2−^ adsorption on chitosan is the electrostatic interaction between the negatively charged anions and the positively charged protonated chitosan amino groups, which is more efficient between pH values of 2 and 4. On the opposite, nutrient desorption occurs at pH >12.^[^ ^227^, ^243^, ^259^ ^]^ Chitosan has also been used for NH_4_^+^ recovery, thanks to the interaction with the hydroxyl groups. Safie et al. compared the ammonium adsorption capacity of chitosan of different molecular weights with those of natural and activated zeolite.^[^ ^242^ ^]^ Starting from a water solution with a concentration of 50 mg L^−1^ NH_4_^+^, the authors reported an adsorption capacity of 0.331 mg g^−1^ for high molecular weight chitosan, 0.769 mg g^−1^ for low molecular weight chitosan, 2.162 mg g^−1^ for natural zeolite and 2.937 mg g^−1^ for activated zeolite. Chitosan can be used as hydrogels, which are easy to apply and have high adsorption performance. Crosslinking can help to overcome instability of chitosan in acidic environments. Jóźwiak et al. examined the adsorption efficiency of chitosan hydrogel beads crosslinked ionically with sodium citrate and covalently with epichlorohydrin.^[^ ^243^ ^]^ The best adsorption capacity was obtained when chitosan was crosslinked with epichlorohydrin with 1.23 mmol g^−1^ P in form of phosphate, 0.94 mmol g^−1^ N in form of nitrite and 0.76 mmol g^−1^ N in form of nitrate. As expected, for both types of hydrogel the performance was the highest at pH 3 and decreased at increasing pH values. Mahaninia et al. crosslinked chitosan with variable amounts (2.5%wt. or 5%wt.) of glutaraldehyde and epichlorohydrin aiming to achieve a more hydrophobic and a more hydrophilic hydrogel, respectively.^[^ ^244^ ^]^ The authors tested the beads for the adsorption of HPO_4_^2−^ at pH 8.5 from an aqueous solution, to investigate the chitosan behavior in alkaline aquatic environments. The study showed that hydrogels with 2.5%wt. crosslinker performed better than the ones with 5%wt., and that the hydrophilicity of epichlorohydrin improved the adsorption capacity of the beads as compared with the more hydrophobic glutaraldehyde. 52.1 mg g^−1^ HPO_4_^2−^ was adsorbed by chitosan crosslinked with 2.5%wt. epichlorohydrin.

The incorporation of fillers into chitosan has been proven to improve its adsorption capacity and reduce its weaknesses, such as mechanical strength and reusability, as compared to the pure polymer.^[^ ^259^ ^]^ Zuo et al. investigated to use of chitosan‐biochar composites with different chitosan loadings followed by ultrafiltration to recover nutrients from potato starch wastewater, a byproduct of potato processing.^[^ ^245^ ^]^ The composite containing 40%wt. of chitosan was most efficient, removing 93.3% ammonium and 91.2% total P from the wastewater. Gaouar Yadi et al. compared the ammonia removal efficiency of pure bentonite with that of bentonite–chitosan and bentonite–chitin composites prepared via coagulation method.^[^ ^246^ ^]^ The best performance was achieved by bentonite–chitosan composites, with an NH_4_^+^ removal capacity of 11.6 mg g^−1^, followed by bentonite–chitin and natural bentonite, which were able to remove 1.04 mg g^−1^ and 0.75 mg g^−1^ ammonium, respectively. The success of the bentonite‐chitosan beads was attributed to their high surface area and porosity. Mondal et al. prepared composites by grafting chitosan onto dried and crushed banana peel, known to be an effective NH_4_^+^ adsorbent.^[^ ^247^ ^]^ The composite showed high adsorption capacity of both ammonium (42.2 mg g^−1^) and phosphate (15.9 mg g^−1^) from urine. The adsorption capacity of chitosan toward phosphate and nitrate can be increased by the incorporation of multivalent cations such as La^3+^ in the biopolymer matrix.^[^ ^259^ ^]^ For instance, a chitosan membrane containing lanthanum, synthesized by Karthikeyan et al., showed adsorption capacity of 76.6 mg g^−1^ and 62.6 mg g^−1^ for phosphate and nitrate ions, respectively.^[^ ^248^ ^]^

Lignin is also studied as an adsorbent for nutrient removal from wastewater. Wang et al. utilized aminated lignin for phosphate recovery from wastewater, and tested the applicability of the recovered P‐loaded aminated lignin as a slow‐release fertilizer via water‐soluble release and soil column leaching experiments.^[^ ^249^ ^]^ The aminated lignin showed a maximum phosphate adsorption capacity of 46.28 mg g^−1^ at pH 6 and displayed efficient slow release of N and P both in water and soil, resulting promising for the application as slow‐release fertilizer. Lignin has also been applied as an adsorbent for nutrient recovery in the form of nanoparticles, which are more efficient than the macroscale lignin thanks to the higher surface area. Li et al. have prepared a nanoadsorbent by grafting poly(ethyleneimine) onto epoxidized lignin followed by coprecipitation with iron.^[^ ^182^ ^]^ After recovering the P, the nanocomposite was retrieved using a magnet, and tested as fertilizer for seed germination and seedling growth. The P‐loaded nanoparticles improved the shoot size of mung bean seedlings. In another study, Li et al., first aminated lignin via the Mannich reaction and then utilized it to incorporate Fe_3_O_4_ nanoparticles.^[^ ^250^ ^]^ The particles showed an adsorption capacity of 43 mg g^−1^ HPO_4_^2−^, and after usage, they were recovered by a magnet, regenerated in HCl solution and reused. The recovery rate gradually decreased after six cycles, due to the lignin degradation and the Fe_3_O_4_ nanoparticle exfoliation.

As previously seen for chitosan, the addition of lanthanum to lignin was proven to increase its phosphate adsorption capacity as compared to the pure polymer. Zhang et al. used coprecipitation to prepare a lanthanum‐modified lignin adsorbent, which displayed outstanding P adsorption capacity (107 mg g^−1^) in a pH range of 2−11.^[^ ^251^ ^]^ The two previously described approaches, including the fabrication of lignin nanoparticles and the inclusion of lanthanum, were combined in a study reported by Zong et al.^[^ ^252^ ^]^ The authors first grafted poly(ethyleneimine) on lignin via Mannich reaction, and then used it to synthesize lanthanum‐loaded lignin nanoparticles. The adsorption capacity of the particles for phosphate resulted in 65.8 mg g^−1^ in the pH range 3–9, i.e. 33 times higher than that of unmodified lignin. After the use, the nano‐adsorbent was regenerated in NaOH solution and reused, showing 86% of the original adsorption capacity after three cycles.

Another natural polymer broadly applied as an adsorbent of nutrients from wastewater is alginate. Isik et al. prepared calcium alginate beads, and tested their ammonium and phosphate removal efficiencies from a synthetic aqueous solution (95% NH_4_^+^ and 88% HPO_4_^2−^), simulated wastewater (91% NH_4_^+^ and 47% HPO_4_^2−^) and real wastewater (41% NH_4_^+^ and 10.5% HPO_4_^2−^).^[^ ^230^ ^]^ As previously seen for chitosan and lignin, the adsorption performance of alginate can be improved by a variety of fillers. Kumar et al. synthesized several composites by incorporating bentonite and hydrotalcite in a sodium alginate matrix, which was then crosslinked with Zr^4+^, Ce^3+^, La^3+^, Ca^2+^ and Mg^2+^.^[^ ^253^ ^]^ The highest phosphate adsorption capacity was achieved by beads containing bentonite and crosslinked with Zr^4+^ (30.2 mg g^−1^), Ce^3+^ (26.1 mg g^−1^), La^3+^ (20.7 mg g^−1^). In another study, Kumar et al. incorporated Fe_3_O_4_ into sodium alginate beads in order to target nitrate and phosphate recovery.^[^ ^239^ ^]^ The authors further improved the system by grafting amine groups on the surface of the beads via both *in situ* precipitation and hydrothermal techniques. This adsorbent composite was able to remove 23.8 mg g^−1^ NO_3_^−^ and 30.1 mg g^−1^ HPO_4_^2−^, and could be efficiently regenerated in NaOH solution up to three times. The same authors have recently published another study where sodium alginate was used for the incorporation of triaminotriazine – functionalized graphene oxide.^[^ ^254^ ^]^ The composite beads exhibited a strongly enhanced adsorption capacity toward nitrate (51.8 mg g^−1^) and phosphate (58.5 mg g^−1^) as compared to the starting materials from which they were assembled.

Examples of synthetic biodegradable polymers that have been used to develop adsorbents for nutrient recovery include poly(vinyl alcohol) and poly(acrylic acid). Ding et al. immobilized calcium silicate hydrate powder within a PVA film combined with NaNO_3_.^[^ ^255^ ^]^ The prepared adsorbent displayed an adsorption capacity of 28.1 mg g^−1^ PO_4_^3−^. Wongniramaikul et al. immobilized calcium silicate hydrate nanoparticles within calcium crosslinked poly(vinyl alcohol) film. The developed adsorbent showed a maximum adsorption capacity of 99 mg g^−1^ PO_4_^3−^. Poly(acrylic acid) hydrogels, prepared by free radical polymerization and inserted in a fixed bed column, were utilized by He et al. to remove ammonium from domestic wastewater.^[^ ^257^ ^]^ The hydrogels displayed an adsorption capacity of 8.8 mg g^−1^ in the pH range 4–9. Poly(acrylic acid) was also used by Guo et al., together with sodium alginate, to achieve a hydrogel containing titanium dioxide that was able to recover ammonium from wastewater with an ammonium adsorption capacity of 113 mg g^−1^.^[^ ^258^ ^]^

### 3.3 Coagulants and Flocculants

Coagulation–flocculation is a simple and cost‐effective technique for removing pollutants and particles from wastewater and can also be applied for P recovery.^[^ ^260^, ^261^, ^262^ ^]^ Chemical coagulants, usually inorganic salts, work by neutralizing particle charges, which helps them cluster together into larger masses that are easier to remove. For the removal of P the most commonly used are FeCl_3_ or Al_2_(SO_4_)_3_,^[^ ^263^, ^264^, ^265^ ^]^ which generate a final product where P is bound to Fe or Al, hence less available for plant uptake and less attractive to be used as fertilizer.^[^ ^266^ ^]^ Chemical flocculants are, instead, organic polymers with large molecular weight and high charge concentrations, which interact with the targeted ions or particles creating bridges between them. This process generates bigger flocs, which are easier to separate.^[^ ^260^, ^261^, ^262^, ^267^ ^]^ Biodegradable polymers represent a sustainable alternative to remove organic and inorganic contaminants via coagulation/flocculation.^[^ ^260^, ^261^, ^262^ ^]^ However, the recovery of nutrients with chitosan, lignin, starch and other polymers derived from natural sources has not been widely reported yet. Only a few examples of studies about phosphorous recovery can be found in the literature, which are listed in **Table** **9**.

**Table 9:** Biodegradable polymers utilized as coagulants/flocculants for recovery of phosphorous.

Polymer	Additive	Preparation method	P removal efficiency [%]	Ref.
Starch	–	–	80	[268]
Starch	–	–	86	[269]
Starch	CTA^a^ ^)^	–	98.7 inorganic P, 97.5 organic P	[270]
Chitosan/starch	Fe_3_O_4_ nanoparticles	Precipitation	93	[271]
Lignin	METAC^b^ ^)^	Polymerization	42	[272]

^a)^ Chloro‐2‐hydroxypropyl trimethyl ammonium chloride (CTA);

^b)^ [2‐(methacryloyloxy) ethyl] trimethylammonium chloride (METAC).

Although starch can be chemically modified to introduce positive or negative charges, making it interesting for its use as a flocculant material,^[^ ^260^, ^261^ ^]^ only a few articles focus on its application for P recovery via coagulation. Righetto et al. tested two starch‐based commercially available coagulants for the pretreatment of wastewater before membrane separation.^[^ ^268^ ^]^ The authors aimed to first remove P via flocculation and later N via membrane separation. A reduction of 18% N and 80% P was achieved with the flocculation process, applied at pH ranging between 8 and 9, and the experimental setup was efficiently converted to a continuous system at pilot scale. Rolf et al. evaluated the removal of P from wastewater derived from pig manure using five different commercial coagulants based on starch, two cationic and three anionic.^[^ ^269^ ^]^ The best candidate allowed the separation of 86% P, while leaving other nutrients such as N and K unchanged. Ren et al. applied five different starch‐based flocculants as assisting agents for FeCl_3_, a traditional inorganic coagulant, to remove P from simulated wastewater.^[^ ^270^ ^]^ The natural‐based flocculants showed high efficiency in removing P (99% inorganic P and 98% organic P) and significantly reduced the required dosage of FeCl_3_.

Sibiya et al. synthesized two magnetized coagulants by co‐precipitation of Fe_3_O_4_ nanoparticles into chitosan and starch.^[^ ^271^ ^]^ The composites were prepared by mixing each of the two the natural polymers with the metal nanoparticles in three different ratios: 1:2, 1:1, and 2:1, being the 1:1 coagulant the one with the best properties, achieving 93% P removal.

Lignin has also been used as a flocculant.^[^ ^273^ ^]^ However, it mainly finds application in the removal of organic and inorganic pollutants, not in nutrient recovery. An interesting exception is a study by Moore et al., who applied radical graft polymerization of [2‐(methacryloyloxy) ethyl] trimethylammonium chloride (METAC) on different industrial lignin types.^[^ ^272^ ^]^ The products were utilized as flocculants for removing, among other compounds, phosphorous from synthetic municipal wastewater. The authors tested the lignin‐based flocculant together with alum, a commonly used inorganic coagulant, and removed 42% P from the substrate.

## 4 Conclusions and Future Perspectives

This review has highlighted the opportunities for biodegradable polymers to accelerate the transition toward a more sustainable agriculture and a more circular use of the plant nutrients, which is essential for global food production and environmental health. The use of slow‐ and controlled‐release fertilizers opens new opportunities for more efficient nutrient delivery, targeting an optimal crop uptake and minimum losses. Our vision is that future research will focus on customizing fertilizing products for the specific soil and plant species for which they will be used. A major importance will be given to the relationship between fertilizer, plant and soil, which is fundamental for plant nutrition and is strongly influenced by the environmental conditions. Particular attention will also be paid to improve water management, as water shortages and drought conditions can result in soil desertification and salinization, threatening both agricultural sustainability and food security.^[^ ^274^, ^275^ ^]^ Superabsorbent polymers can be key materials to ameliorate the use of water in agriculture, retaining moisture in the soil and reducing the irrigation water consumption.^[^ ^276^, ^277^ ^]^ As reported in this review, polymer‐based hydrogels are especially strategic for their water retention capacity, allowing a very efficient use of water. The hydrophilic natural biodegradable polymers herein described are promising candidates for this type of application, but their sensitivity to microbes and weak mechanical properties can reduce the efficacy in slowing down and controlling the release of nitrogen and phosphorous in soil.^[^ ^33^ ^]^ Mixing them with synthetic polymers, more durable and resistant, is a clever way to achieve more efficient fertilizers.^[^ ^278^ ^]^ However, the fate of these synthetic materials is still a major concern for environmental safety. To address this challenge, a further effort must be dedicated to analyzing in detail the degradation products of multiple synthetic biodegradable polymers under different soil conditions like temperature and moisture. Future studies should also consider more deeply the effect of soil microbiome on polymer degradation, as well as the effect of polymer degradation on soil and water environmental parameters. More research work should be focused on the generation and migration of degradation‐derived pollutants in soil and on the relative food contamination. Another important area of application for biodegradable polymers is nutrient recovery from wastewater. Efficient nutrient recovery processes will help to reduce water pollution and provide potential recycled fertilizers that can diminish the dependency on the Haber‐Bosch process and on critical phosphate rock. Each nutrient recovery process reaches optimal efficiency when applied in a certain condition and within a specific nutrient concentration. Therefore, no single technique will provide a comprehensive solution to closing the N and P cycle. The trend should hence be the integration of novel technologies to implement nutrient recovery. Moreover, the research works where nutrients are recovered and then directly recycled as fertilizers are still very few, so there is a wide gap in the literature to be filled with new technologies able to close the nutrient loop and efficiently reuse the waste N and P.

## Conflict of Interest

The authors declare no conflict of interest.
